# Polymorphism and Mechanochromism in 2-Phenylbenzothiazole Cyclometalated Pt^II^ Complexes with Chelating N^∧^O Ligands

**DOI:** 10.1021/acs.inorgchem.2c03423

**Published:** 2022-11-28

**Authors:** David Gómez de Segura, Elena Lalinde, M. Teresa Moreno

**Affiliations:** Departamento de Química-Centro de Síntesis Química de La Rioja (CISQ), Universidad de La Rioja, 26006 Logroño, Spain

## Abstract

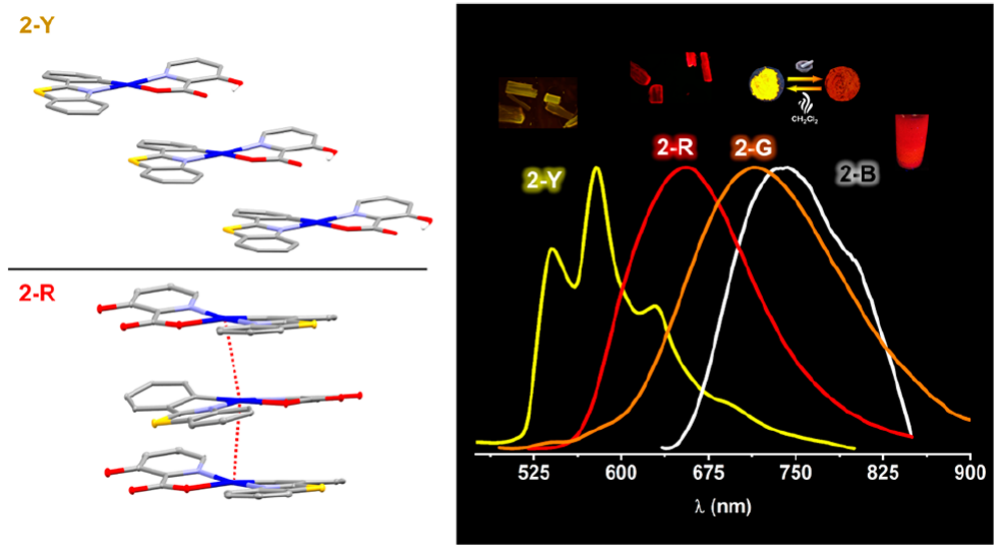

New cyclometalated Pt^II^ complexes with 2-phenylbenzothiazole (pbt) and two different picolinate ligands [Pt(pbt)(R-pic-κ*N,O*)] (R = H (**1**), OH (**2**)) were prepared. In contrast to **1**, the OH substituent group on **2** allows modulation of the packing in the solid state through donor–acceptor H-bonding interactions with the CH_2_Cl_2_ solvent. Thus, three pseudopolymorphs of **2** with different aggregation degrees were isolated, including yellow **2-Y**, orange-red **2-R** (**2·0.5CH**_**2**_**Cl**_**2**_) and black **2-B** (**2·0.75CH**_**2**_**Cl**_**2**_) with emissions at 540, 656, and 740 nm, respectively, in the solid state at 298 K. **2-R** and **2-B** can be transformed to the pristine solid **2**. Studies of their crystal structures show that **1** and **2-Y** stack in columns with only π···π stacking interactions, whereas **2-R** displays strong aggregated 1D infinite chains based on Pt···Pt and π···π stacking interactions, consistent with the colors and the photophysical properties, measured in several media. Interestingly, **1** and **2** exhibit reversible mechanochromic behavior with high contrast in the color and color emission upon mechanical grinding due to a phase transition between a crystalline and an amorphous state, as confirmed by powder X-ray diffraction (PXRD) studies. Theoretical calculations indicate that Pt···Pt contacts are more relevant in the trimers and tetramers than in the dimers, particularly in their T_1_ states, associated with a change from a ^3^IL/^3^MLCT transition in the monomer to ^3^MM(L+L′)CT in the oligomers. Noncovalent interaction (NCI) theoretical studies indicate that the π···π stacking among chelates also exerts a strong influence in the metal-metal-to-ligand charge transfer transition character.

## Abstract 2

A combined structural, photophysical, and computational analysis of the interaction topology of two cycloplatinated(II) complexes based on pbt and picolinate chelate ligands is presented. Both complexes exhibit reversible mechanochromic behavior with high-contrast color emission changes.

## Introduction

Phosphorescent cyclometalated Pt^II^ complexes are of crucial importance in applications such as emitting devices,^1^ chemosensors,^2^ sensitizers,^3^ biological cell imaging,^4^ and photocatalysts.^5^ These platinum(II) chromophores exhibit high-efficiency photoluminescence (PL) quantum yields (ϕ) and lifetimes (τ), as well as remarkable changes in emission color depending on the electronic properties of the cyclometalated and auxiliary ligands. Typically, the lowest-energy excited state is characterized as metal-to-ligand charge transfer (^3^MLCT), ligand-to-ligand charge transfer (^3^LLCT), and/or ligand-centered (^3^LC) triplet character,^6^ favored by the rigidity and strong ligand field of the cyclometalated ligand and the very fast singlet–triplet intersystem crossing (ISC) provoked by the 5d platinum atom.

Furthermore, these complexes are significantly assembled in distinct stacking arrangements through Pt···Pt and/or π···π interactions, accounting for the wide ranges of color and emission observed from these aggregates. These interactions become particularly relevant in the solid state and in concentrated solutions, with additional photophysical features such as metal-metal-to-ligand charge transfer (^3^MMLCT) transitions and/or ^3^ππ* (excimers or aggregates), which result in broad and red-shifted emissions in comparison to the monomers. Excellent reviews concerning this topic and the consequences on the absorption and emission properties have been recently published.^7^ Particularly, polymorphs of the same species with distinct Pt···Pt and/or π···π interactions can show distinctive absorption and emission colors by changing the molecular stacking.^8^ In addition, noncovalent intermolecular interactions such as H bonds, halogen bridges, van der Waals, and others have been observed in some cyclometalated platinum(II) complexes, leading to aggregation-induced emission (AIE).^7d,9^ However, in some cases the ^3^ππ* excimer emission can be quenched in the aggregated state, a phenomenon usually defined as aggregation-induced quenching (AIQ).^10^ All of these interactions are usually sensitive to external stimuli, such as VOCs, solvents, pH, temperature, and pressure, being of interest for the design of functional materials.^8c,11^ In particular, the number of reported pressure-sensitive luminescent compounds in the last years is relevant, with examples based on cyclometalated platinum complexes.^8b,8,11c,11d,12^ Among them, a few AIE-active complexes with mechanochromic properties have been reported.^8b,12b,13^

In this context, some mononuclear cycloplatinated (C^N) complexes featuring N^O chelating auxiliary ligands have been studied.^8d,9e,14^ Most of the reported complexes adopt a *tran*s-N,N configuration around the Pt^II^ center,^8d,9a,9e,14a−14c,14f,14h^ with only a few having a *cis*-N,N configuration.^14d,14e^ Within the framework of our recent studies dealing with the photophysical studies on Pt^II^, Pt^IV^, and Ir^III^ complexes with phenylbenzothiazol (pbt)-based cyclometalated ligands,^15^ we now describe the structures, optical properties, and computational analysis of two new cycloplatinated(II) complexes featuring pbt and picolinate ligands, [Pt(pbt)(R-pic-κ*N,O*)] (R = H (**1**), OH (**2**)), including three distinct pseudopolymorphs for **2**. Importantly, complexes **1** and **2** exhibit a reversible phosphorescent mechanochromic phenomenon with a striking contrast in luminescence colors.

## Results and Discussion

### Synthesis and Characterization

**Scheme 1:**
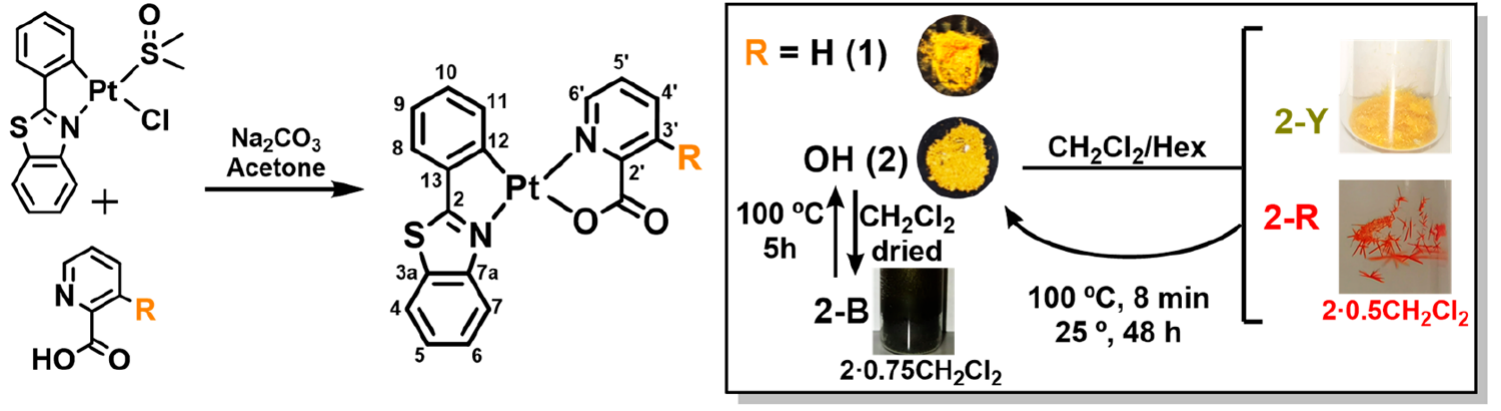
Labeling and Synthesis Conditions for **1** and **2** in Their Different Polymorphic Forms

The synthesis of the complexes [Pt(pbt)(R-pic-κ*N,O*)] (R = H (**1**), OH (**2**)) (Scheme 1) was carried out by reaction of the solvate [Pt(pbt)Cl(DMSO)]^15d^ with the corresponding picolinic acid (1:1), in the presence of excess of Na_2_CO_3_, in acetone at room temperature (see the Experimental Section). Both complexes were isolated by conventional procedures as yellow solids in high yield. Both derivatives (**1** and **2**) exhibit one C=O stretching band at 1660 (**1**) and 1641 cm^–1^ (**2**) and one C–O band at 1336 (**1**) and 1326 cm^–1^ (**2**) and also the characteristic ν(OH) band at 2695 cm^–1^ for **2**. Their MALDI-TOF(+)-MS spectra show in both complexes the molecular peak [M]^+^ as the parent peak. The ^1^H and ^13^C{^1^H} NMR spectra show the expected integration of the pbt cyclometalated and picolinate ligands in a 1:1 ratio, and the signals were assigned on the basis of ^1^H–^1^H and ^13^C–^1^H correlations (see the Experimental Section and Figures S1 and S2). In their ^1^H NMR spectra, the most deshielded signal is the pbt H^7^ proton (δ 9.30 (**1**), 9.14 (**2**)), which appears as a doublet, and the H^6′^ proton of the picolinate (δ 9.26 (**1**), 8.74 (**2**)), which shows platinum satellites (^3^*J*_Pt–H_ ≈ 45 Hz). The ^13^C{^1^H} NMR spectrum of **1** displays, as the most deshielded signals, those corresponding to the C^2^ (δ 181.6) and carboxylic carbons of the picolinate ligand (δ 172.9). These complexes are only soluble in CH_2_Cl_2_ and CHCl_3_ and partially in acetone or THF. Interestingly, we found that when a CH_2_Cl_2_ solution of **2-Pristine** was slowly dried in air, a black solid was obtained (phase **2-B**). Unfortunately, all attempts to obtain crystals of this black phase **2-B** were unsuscefull. By NMR spectroscopy, this solid (**2-B**) is seen to incorporate 0.75 molecules of CH_2_Cl_2_ per molecule of **2** (**2·0.75CH**_**2**_**Cl**_**2**_, Figure S3). Notwithstanding, two pseudopolymorphs of **2**, a yellow (**2-Y**) and a red form (**2-R**), could be simultaneously obtained by crystallization from CH_2_Cl_2_/*n-*hexane. The red form, **2-R**, incorporates 0.5 molecule of CH_2_Cl_2_ to the lattice as **2·0.5CH**_**2**_**Cl**_**2**_ (confirmed by X-ray studies (see below) and by NMR spectroscopy (Figure S4)), which is easily lost in 8 min in the oven at 100 °C or in 48 h at room temperature, reverting to the unsolvated yellow phase (Figure S5). The black pseudopolymorph (**2-B** phase) reverts to a yellow solid after 5 h in the oven at 100 °C, also suggesting a structural transformation to the pristine unsolvated phase.

### Crystal Structure Analysis

**Figure 1:**
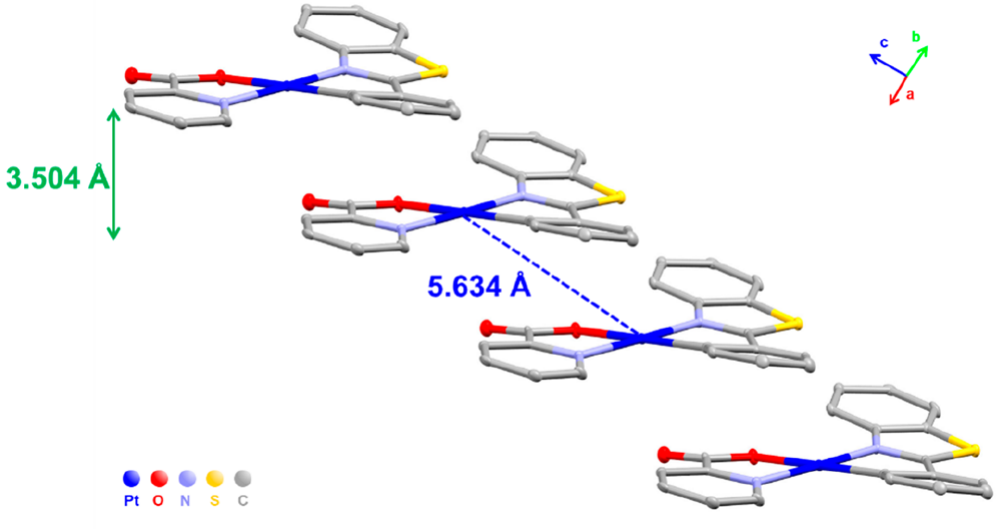
Crystal stacking in the structure of **1** along the *c* axis.

Yellow-emissive microcrystals of **1** were obtained by slow diffusion of *n-*hexane into a saturated solution of **1** in acetone at room temperature (Figure 1, Figure S6, Table 1, and Tables S1 and S2). Attempts to obtain crystals from CH_2_Cl_2_ solvent (as **2**) were unsuccessful. The X-ray diffraction structure confirms its *trans-*N,N configuration, in which the N atom of the pic ligand is coordinated *trans* to the N atom of the pbt ligand. The almost planar molecules are stacked into a 1D stairlike columnar fashion along the *c* axis, with a slipped head-to-head arrangement through π···π stacking interactions, the shortest interplanar distance being 3.504 Å between the phenyl ring of the pbt and the heteroatomic ring of the pic ligand. However, the Pt···Pt distance is rather long, 5.634 Å (longer than twice the van der Waals radius of Pt of 3.50 Å),^16^ excluding Pt···Pt interactions.

**Table 1:** Color, Emission Color, and Selected Distances (Å) of Crystals **1**, **2-Y**, and **2-R**

	**1**	**2 (2-Y)**	**2·0.5CH**_**2**_**Cl**_**2**_**(2-R)**
color	yellow	yellow	orange-red
emission	yellow	yellow	red
Pt(1)–C(1) (Å)	2.020(8)	2.007(3)	2.002(6)
Pt(1)–N(1) (Å)	2.010(6)	2.028(3)	2.011(5)
Pt(1)–N(2) (Å)	2.041(6)	2.042(3)	2.021(5)
Pt(1)–O(1) (Å)	2.118(6)	2.113(3)	2.129(4)
Pt···Pt (Å)	5.634	5.426	3.387_dimer A_/3.726
			3.411_dimer B_/3.701
*d*_interplanar_ (Å)	3.504	3.660	3.205_dimer A_/3.270
			3.302_dimer B_/3.276

**Figure 2:**
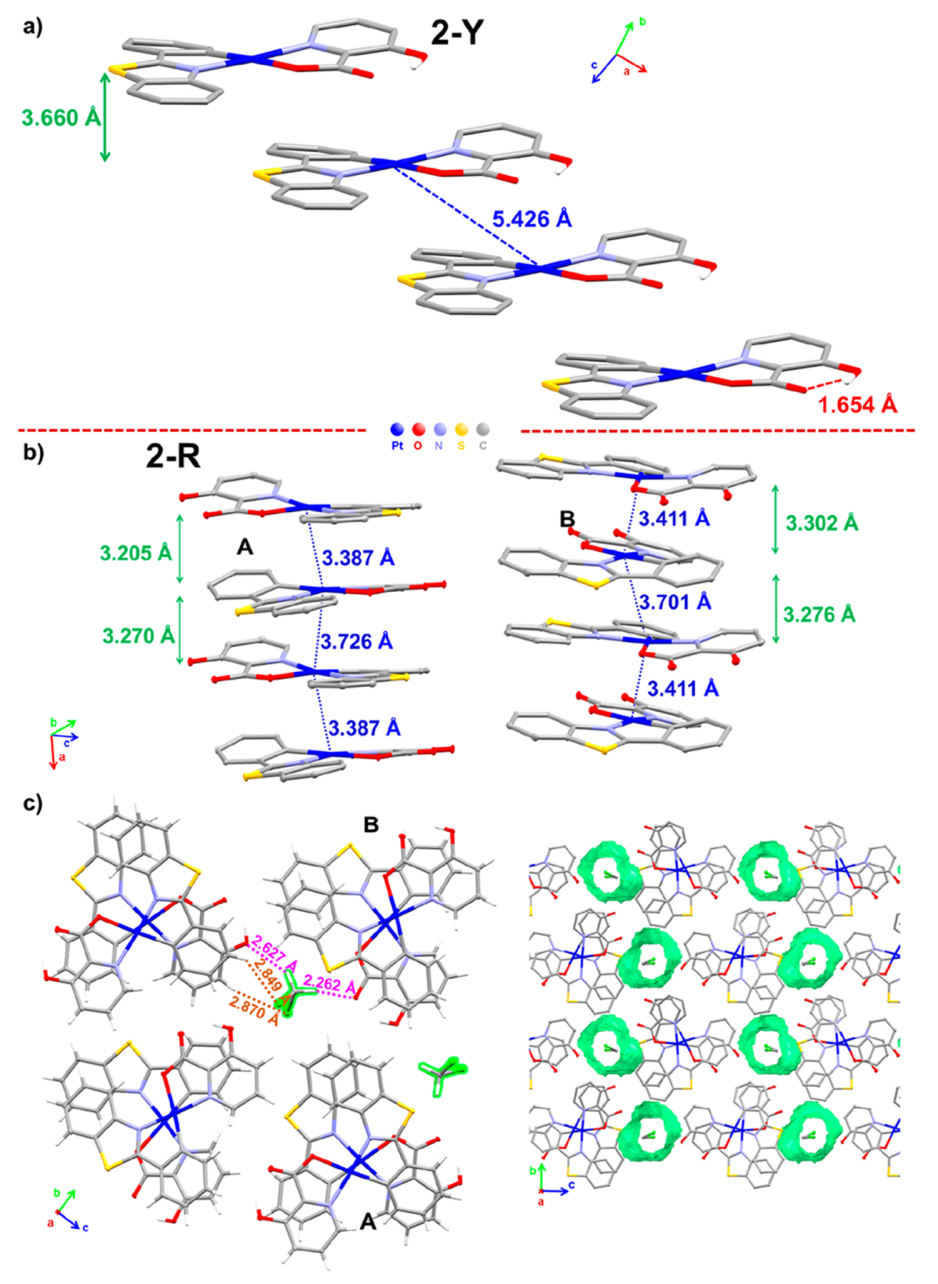
Crystal stacking in the structures of (a) **2-Y** along the *c* axis and (b) **2-R** (molecules A and B) along the *a* axis, showing the π···π interplanar and Pt···Pt distances and (c) top view from the *a* axis of the chains of **2-R** (molecules A and B) showing the CH_2_Cl_2_ solvent localization and contacts.

Yellow (**2-Y**) and red (**2·0.5CH**_**2**_**Cl**_**2**_) needles were simultaneously grown by slow diffusion of *n-*hexane into a CH_2_Cl_2_ solution of **2** at 298 K. Notwithstanding, yellow monocrystals were also obtained from CHCl_3_/*n-*hexane with crystallographic data similar to those of **2-Y**. Yellow emissive crystals of **2-Y** show a molecular packing similar to that shown for **1**, based on a stairlike stacking structure, the shortest π···π stacking distance being 3.660 Å with a Pt···Pt separation of 5.426 Å (Figure 2a, Figure S7, Table 1, and Tables S1 and S2). In the molecule, the Pt–C and Pt–N bond distances are comparable to those in **1**, indicating the small influence of the OH group in the structure. As expected from the hydrogen-bonding ability, an intramolecular hydrogen bond between the carboxylate and the OH^–^ substituent of each picolinate ligand (O–H···O 1.654 Å; O–H–O 148.34°) was formed.

On the other hand, the crystal structure of the red-emissive red crystals (**2-R**) was significantly different from that of **2-Y**. In the asymmetric unit, two very similar head-to-tail dimers (**A** and **B**) and two molecules of CH_2_Cl_2_ are found. Each dimer stacks into infinite staggered columns in the crystal packing (Figure 2b). Within individual columns, neighboring molecules have an antiparallel arrangement with alternating Pt···Pt distances of 3.387 (dimer A)/3.726 Å (interdimer) and 3.411 (dimer B)/3.701 Å (interdimer) and notably short (3.205–3.302 Å) interplanar distances. The short Pt···Pt distances in the unit dimer imply strong Pt···Pt interactions, which account for the lower-energy emission of the crystals **2-R** in relation **to 2-Y** (see discussion below). The molecules stack along the *a* axis in a columnar structure with a Pt–Pt–Pt angle of ∼155.15° and O–Pt–Pt–O torsion angles of 60.7 (dimer A) and 57.2° (dimer B). The presence of CH_2_Cl_2_ solvent molecules creates a continuous channel along the *a* axis, running parallel to the Pt–Pt stacking, supported by C–H_pic_···Cl (2.870, 2.849 Å) and C–O_hydroxyl_···H_CH2Cl2_ (2.627 Å) hydrogen contacts (Figure 2c and Figure S8). The donor–acceptor hydrogen bond between the OH substituent and the CH_2_Cl_2_ solvent plays a key role in the stabilization of this pseudopolymorph. The total solvent volume occupies 11.7% of the total volume of the unit cell (408.8 Å^3^ occupied by CH_2_Cl_2_ in the unit cell).

**Figure 3:**
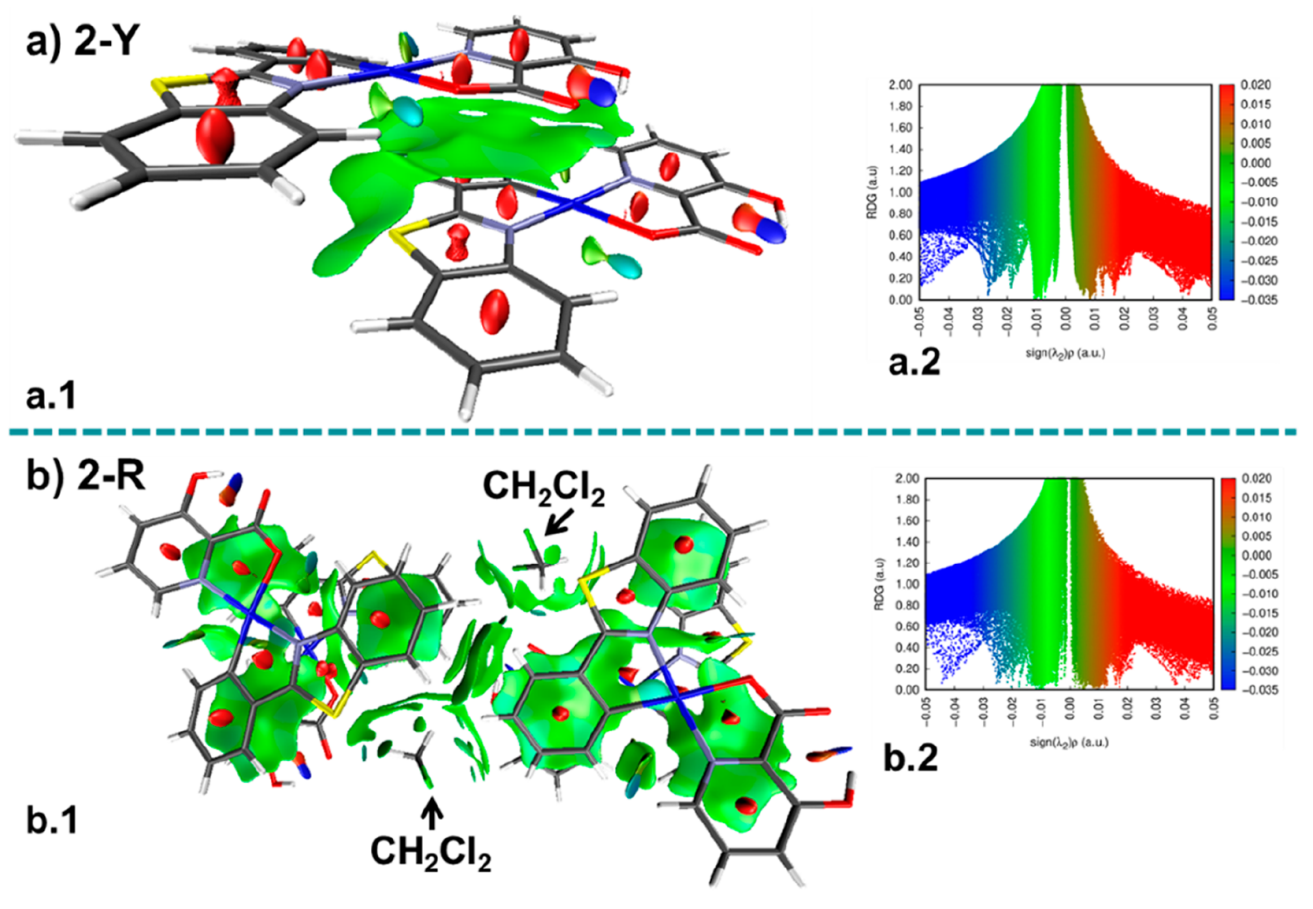
NCI analysis for (a) **2-Y** and (b) **2-R** crystal structures (isovalue 0.3 au).

To get insight from the study of the stacking structures of **2-Y** and **2-R**, we have carried out the computational analysis of noncovalent interactions (NCIs) based on the promolecular density of the X-ray structures. NCI surface plots show a spatial distribution and identification of the noncovalent interactions in the real space. These contacts can be classified by the peaks that emerge in the reduced density gradient (RDG) at low electronic density values.^17^ A qualitative classification of the noncovalent interaction type can be predicted by the value of the product of the electron density (ρ) and the sign of the second Hessian eigenvalue (λ_2_), (sign(λ_2_)ρ). Red isosurfaces and repulsive interactions are associated with a positive sign of the second Hessian eigenvalue (λ_2_ > 0). Values of λ_2_ close to zero indicate van der Waals weak contacts, and the surface is shown in green. Finally, negative values of λ_2_, associated with blue or blue-green surfaces, suggest attractive noncovalent interactions. As is shown in Figure 3, the **2-Y** structure (Figures 3a1) shows only weak π···π interactions between a pbt and a picolinate ligand of different molecules, whereas **2-R** (Figure 3b1) exhibits extended green regions associated with π···π contacts between face-to-face pbt and picolinate ligands, indicative of greater contacts. In addition, small green bluish disks along the Pt···Pt···Pt axis support attractive platinum–platinum interactions, and those associated with intermolecular hydrogen contacts with the CH_2_Cl_2_ solvent are also clearly visible (Figure 3b1).

### Photophysical Properties and Theoretical Calculations

#### Absorption Properties in Solution and TD-DFT Calculations

**Figure 4:**
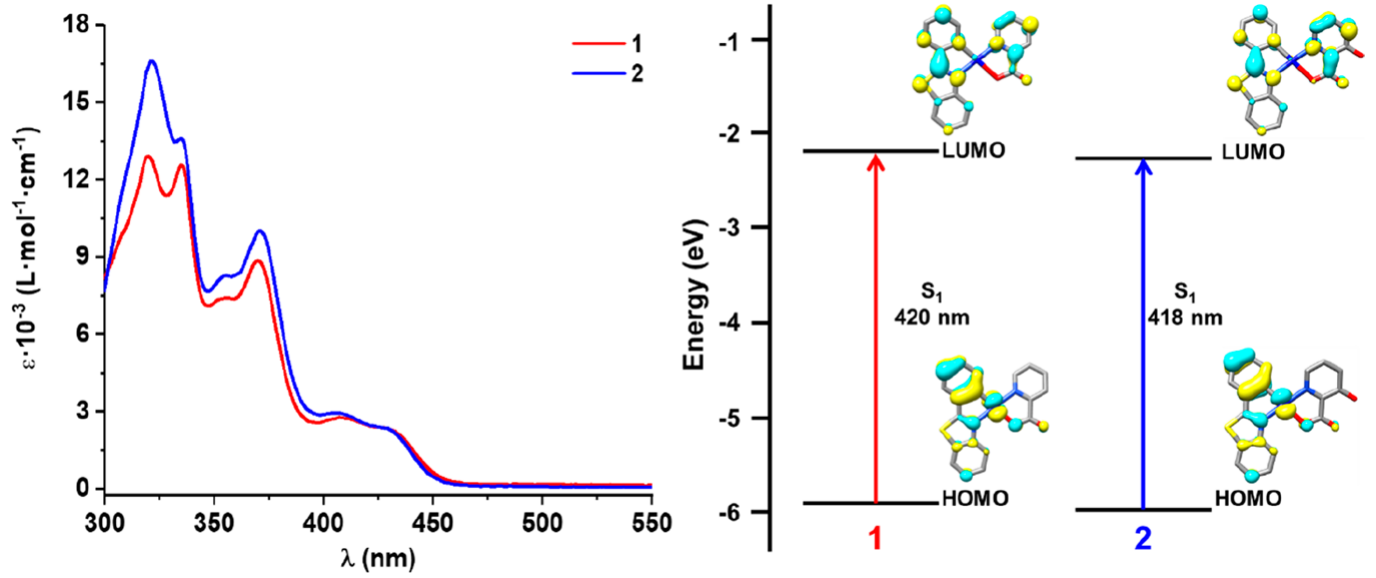
UV–vis absorption spectra of **1** and **2** in CH_2_Cl_2_ (5 × 10^–5^ M) and schematic representations of the HOMO and LUMO.

The UV–vis absorption spectra of the complexes were collected in CH_2_Cl_2_ solution (5 × 10^–5^ M) at 298 K (Figure 4 and Table S3), and assignments were done from TD-DFT calculations in CH_2_Cl_2_ (PCM model) (see details in the Supporting Information, Tables S4 and S5, and Figure S9). The calculated bond distances and angles agree with those of the crystal structure data (Tables S6 and S7), which ensure the accuracy of the DFT calculations at the B3LYP level. The high-energy intense absorption bands (λ < 350 nm, ε = 10^4^ M^–1^ cm^–1^) are mainly attributed to intraligand ^1^IL (π–π*) transitions located on the cyclometalated pbt ligand, with a contribution of ^1^(M+L)L′CT (L′ = pic). The absorptions at around 370 nm also have the participation of both chelating ligands, being attributed to a mixed charge transfer ^1^M(L+L′)CT/^1^LL′CT character. The characteristic low-energy features, with lower extinction coefficients, are similar in shape and energy in both complexes (431 (**1**), 429 nm (**2**)), suggesting that the hydroxy substituent of the picolinate ligand has a weak influence on the transition. TD-DFT calculations for both complexes indicate that the lowest S_1_ state (420 (**1**) and 418 nm (**2**)) is contributed by a HOMO to LUMO transition (97% (**1**) and 96% (**2**)) (Tables S4 and S5, Figure 4, and Figure S9), the HOMO being located on the pbt (58% (**1**) and 63% (**2**)) with a notable contribution from Pt (34% (**1**) and 32% (**2**)) and reduced on the coligand N^O (8% (**1**) and 5% (**2**)) and the LUMO being located on the pbt (63% (**1**) and 56% (**2**)) and the N^O picolinate ligand (32% (**1**) and 39% (**2**)). According to these theoretical calculations, the low-energy absorption can be attributed to an intraligand-based transition ^1^IL centered on the cyclometalated pbt ligand with contribution of charge transfer from the metal to both chelating ligands ^1^M(L+L′)CT (L = pbt, L′ = N^O).

#### Emissions in Film and Solution and DFT Calculations

**Figure 5:**
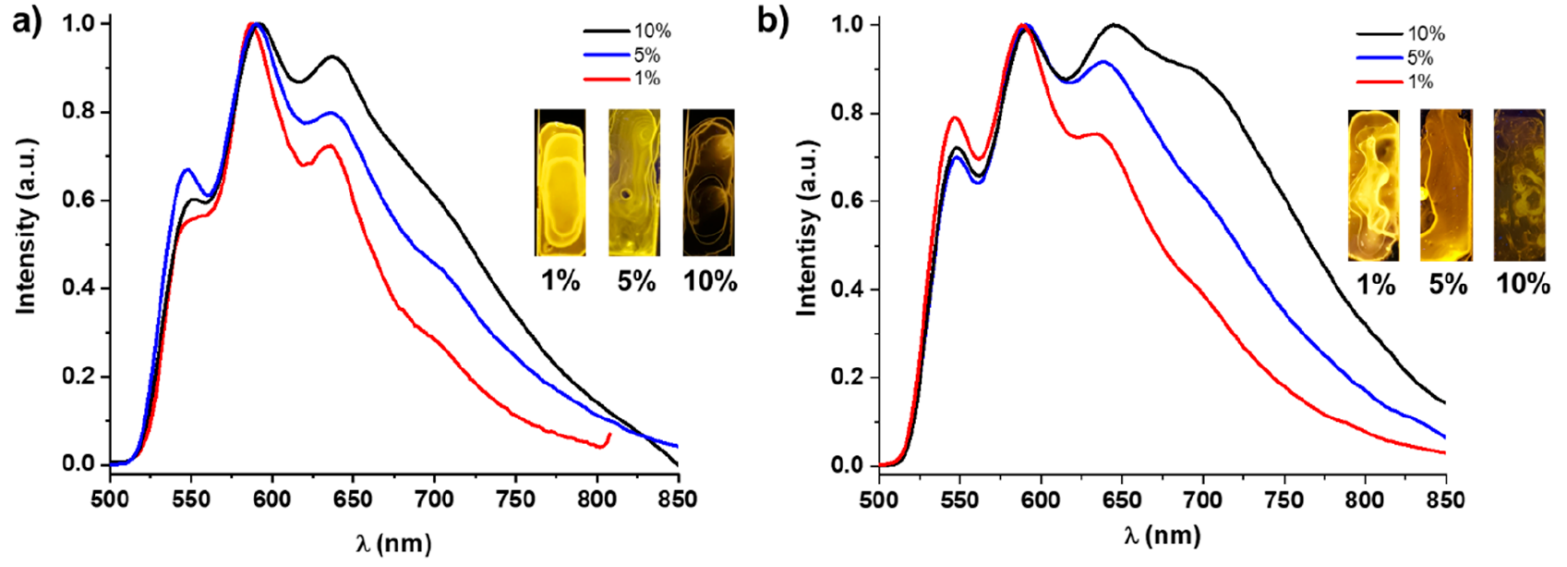
Emission (λ_ex_ ≈ 440 nm) of (a) **1** and (b) **2** in PS film at 1, 5 and 10 wt %.

In this section we present the emission properties of both complexes in doped polystyrene films (PS 1–10 wt %) and in solution (CH_2_Cl_2_ and THF, 298 and 77 K) (Table S8). Complex **1** exhibits in PS film (1, 5 and 10 wt %) a structured phosphorescence band attributed to the monomer emission (λ_em_ 546 nm; Figure 5a), with a tail that becomes broader at a higher doping concentration. Complex **2** shows a similar emission in films at 1 and 5 wt %, and by increasing the concentration to 10% a broad unstructured band at 720 nm, characteristic of aggregates formed by Pt···Pt and/or π···π stacking interactions, is developed (Figure 5b). The lifetimes fit to two components with average values from 3.9 to 7.3 μs in the band of the monomer, showing a decreasing tendency with an increase in the doping percentage (7.3 μs 1%, 6.7 μs 5% and 4.8 μs 10% (**1**); 6.8 μs 1%, 6.1 μs 5% and 3.9 μs 10% (**2**)), and a τ_av_ value of 7.4 μs for the aggregate band of **2** in concentrated film (10 wt %). The quantum yields increase 3.3- (**1**) and 2.6-fold (**2**) in diluted films in relation to concentrated films, indicating that the aggregation phenomena in these complexes induce emission quenching.

**Figure 6:**
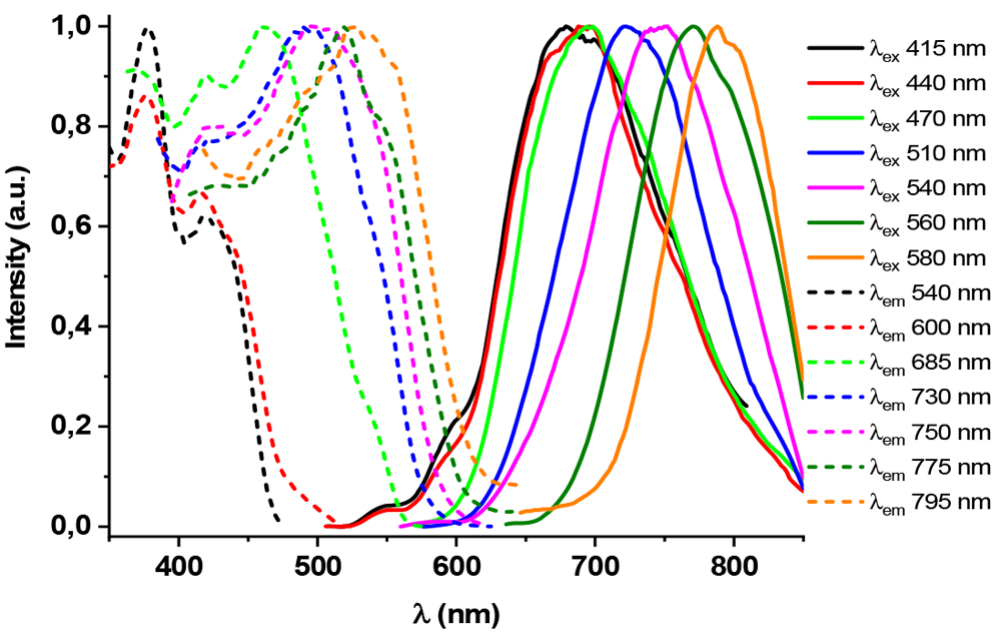
Excitation and emission of **1** in CH_2_Cl_2_ 10^–3^ M at 77 K.

In diluted CH_2_Cl_2_ or THF solution (5 × 10^–5^ M) at 298 K, the complexes display a structured monomer emission (λ_max_ ≈ 540 nm) with quantum yields of 14% (**1**) and 11% (**2**) in THF and 7% in CH_2_Cl_2_ in degassed solutions, decreasing considerably in oxygenated solutions (∼3%) and with lifetimes of ∼13 μs in both CH_2_Cl_2_ and THF (Table S8). This means that the variation of the picolinate ligand or the solvent has a negligible influence on the emission in solution. At room temperature, the emission is not concentration dependent in CH_2_Cl_2_ (up to 10^–3^ M **1**, 5 × 10^–4^ M **2**) (Figure S10). However, in glassy solutions (77 K) both complexes display, in addition to the structured band corresponding to the monomer, low-energy broad bands attributed to the formation of ground-state aggregates. Thus, complex **1** exhibits in dilute glassy solutions (5 × 10^–4^ or 5 × 10^–5^ M, Figure S11) the structured band of the monomer at ∼540 nm and a broad low-energy manifold (∼680 nm CH_2_Cl_2_, 650–680 nm THF) with excitation profiles dependent on the emission wavelength monitoring. By increasing the concentrations to 10^–3^ M in CH_2_Cl_2_, a new broad unstructured band with maxima from 700 to 800 nm, depending on the λ_ex_ used, was observed (Figure 6), indicating the formation of emissive aggregates with distinct Pt···Pt and/or π···π interactions. In the case of the less soluble compound **2**, monomer (∼540 nm) and aggregate emissions (645 nm) are seen in dilute glassy CH_2_Cl_2_ solution at 5 × 10^–5^ M (Figure S12). At 10^–4^ M a red-shifted emission band appears corresponding to different aggregates (760 nm). However, in THF glasses (5 × 10^–5^ M), the presence of the monomer is dominant (Figure S12b).

**Figure 7:**
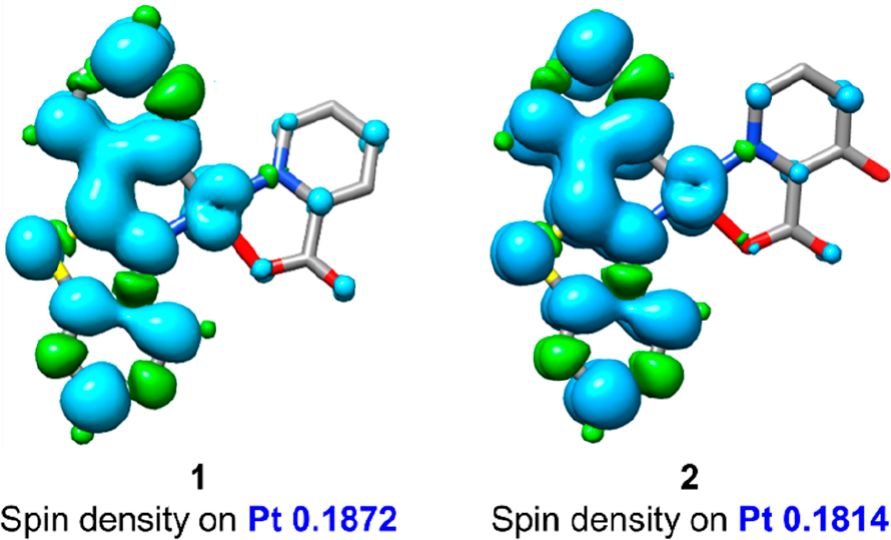
Spin distribution for the lowest triplet excited states in **1** and **2**.

The nature of the monomer emissions was examined through the calculation of the spin density distribution for the triplet excited state (T_1_) based on its corresponding optimized T_1_ geometry. The calculated spin density distribution at the optimized T_1_ state (Figure 7) is mainly located on the pbt ligand and to a lesser extent on the platinum (Pt ∼0.18), with negligible contribution of the N^O coligand, thus supporting an ^3^IL/^3^MLCT state with predominant ^3^IL character. The calculated contribution on the Pt center in the SOMO-1 (Pt 15%) and of the picolinate in the SOMO (N^O, 3% (**1**), 5% (**2**)) decrease in the T_1_ state in relation to the optimized S_0_ geometry (HOMO, Pt 34% (**1**) and 32% (**2**); LUMO, N^O 32% (**1**) and 39% (**2**)), suggesting a notable distortion upon excitation (Table S9). As expected, the computed emission wavelength in CH_2_Cl_2_ (648 (**1**) and 650 nm (**2**)) shows an overestimated value in relation to the observed emission (∼540 nm, CH_2_Cl_2_, 298 K).

#### Emission, Absorption, and Mechanochromism in the Solid State

The solid-state photophysical characteristics of **1** and **2** were evaluated and are compiled in Table 2 (emission) and Table S3 (absorption spectra calculated from their reflectance spectra). Pristine yellow solids **1** and **2** display a structured yellow emission band (λ_max_ 543 (**1**), 545 nm (**2**)) that is slightly red-shifted at 77 K (see Table 2), originating from the intramolecular ^3^IL/^3^MLCT excited state. Notably, both complexes show mechanochromic behavior. The observed responses are quite similar; thus, we present in this section the results of **2**, while the mechanochromism of **1** is detailed in the Figure S13.

**Table 2:** Photophysical Data for **1** and **2** in the Solid State at 298 and 77 K

	298 K			77 K	
compound	λ_em_ (nm) (λ_ex_ (nm))	ϕ	τ (μs)	λ_em_ (nm) (λ_ex_ (nm))	τ (μs)
**1-Pristine**	543, 587, 639 (443)	0.01	12.5	558, 602, 640, 660 (428)	3.2 (92%); 11.1 (8%)
**1-Ground**	700 (538)	0.03	8.5	725 (536)	1.8
**1-Ground** + CH_2_Cl_2_	550, 588, 640 (420)	0.03	10.9	564, 608, 658 (420)	2.8 (58%); 12.2 (41%) [560] 3.8 (68%); 12.9 (32%) [660]
					
**2-Pristine**	545, 580, 630 (418)	0.01	13.6	557, 600, 647 (415)	3.5 (83%); 10.2 (17%)
**2-Ground**	725 (490); 745 (610)	0.06 (490), 0.04 (610)	12.7	568, 605, 700 (463)	2.5
**2-Ground** + CHCl_3_	545, 580, 630 (420)	0.02	13.2	550, 600, 645 (420)	5.9
**2-Y**	540, 578, 630 (420)	0.05	18.7	553, 595, 648 (420)	4.8 (65%); 11.8 (35%)
**2-R**	656 (500)	0.19	6.1	680 (500)	4.1
**2-B**	740 (615)	0.01	12.8	818 (650)	0.7 (68%); 4.7 (32%)

**Figure 8:**
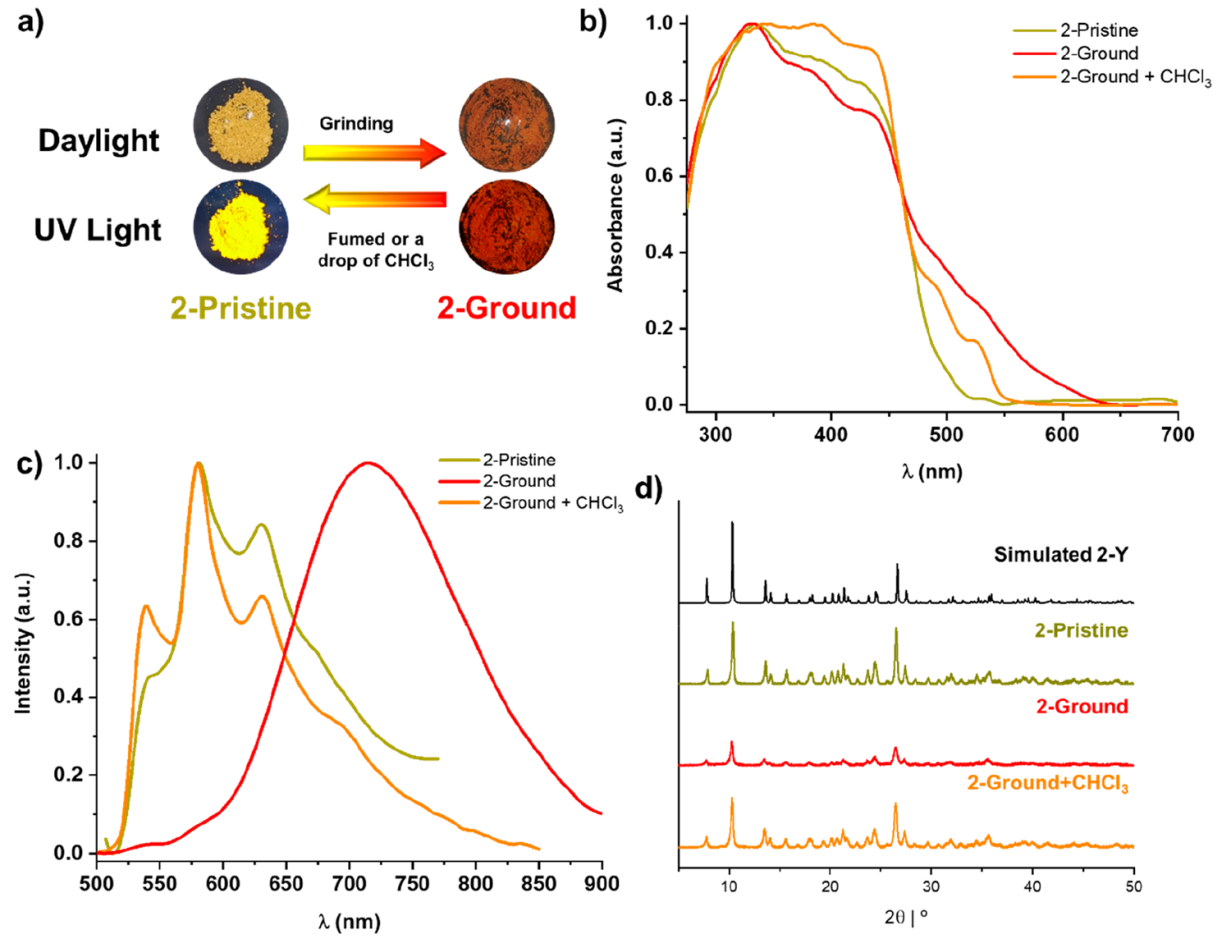
Reversible mechanochromism of **2**: (a) color changes of **2** by grinding and after the addition of a drop or vapors of CHCl_3_ to the ground solid (daylight and UV Light (λ_ex_ 365 nm)); (b) normalized absorption spectra calculated from the reflectance spectra in the solid state; (c) normalized emission spectra of **2-Pristine** powder (λ_ex_ 448 nm), after grinding (λ_ex_ 475 nm), and after the addition of one drop of CHCl_3_ (λ_ex_ 450 nm) at 298 K; (d) changes in the PXRD patterns by mechanical grinding and addition of CHCl_3_ for **2**.

When **2-Pristine** solid is ground in the mortar, its color changes from yellow to orange and the luminescence from yellow to orange-red (Figure 8a,c). The resulting phosphorescence spectra change from a structured band at 545 nm to a broad and structureless low-energy emission band whose maximum ranges from 700 to 745 nm, depending on the excitation wavelength (Figure S16). With reference to previous mechanical-grinding-triggered luminescence switching concerning cyclometalated platinum(II) complexes,^8b,8,11c,11d,12^ the low-energy emission of the ground phase is attributed to the switching on of the ^3^MMLCT excited state. When a few drops or vapors of CHCl_3_ were added to the phase **2-Ground**, the pristine absorption and emission spectra were easily recovered, thus revealing that the complex exhibited reversible mechanochromic behavior. The yellow sample **2-Pristine** exhibits an absorption onset extended to ∼500 nm, while the **2-Ground** sample appears orange, showing an absorption profile extending to ∼630 nm (Figure 8b). The solid-state emission of the ground sample displays an improved quantum yield (ϕ = 6%) together with a slightly shortened radiative lifetime (τ = 12.7 μs) in relation to the pristine solid **2** (ϕ = 1%; τ = 13.6 μs), both indicative of the MMLCT spectral characteristics. The powder X-ray diffraction pattern of **2-Pristine** coincides well with the simulated powder pattern of the crystals of **2-Y** (Figure 8d). Grinding of the yellow pristine material produces a rapid color change to orange and partial loss of crystallinity, showing a great decrease in the peak’s intensity. The amorphous form **2-Ground** created by mechanical force favors the MMLCT, leading to the color change. Upon fuming the amorphous phase **2-Ground** with CHCl_3_ vapors, the color turns back to yellow quickly, partially recovering the pattern of the yellow pristine form (**2-Pristine**). The reversible mechanochromic behavior of **2** was repeated for six cycles without chemical degradation (Figure S17).

**Figure 9:**
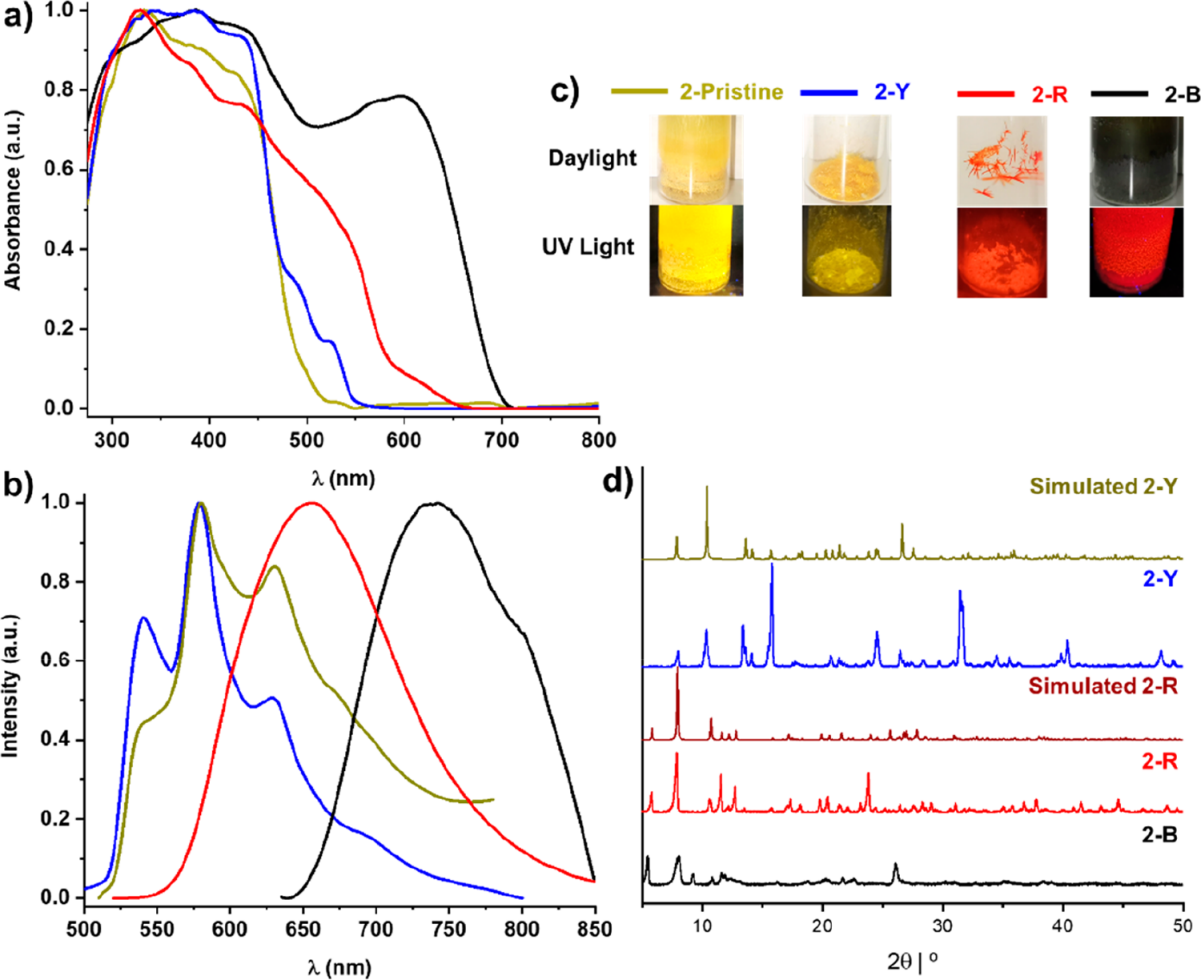
Different pseudopolymorphs of **2**: (a) normalized absorption spectra calculated from their reflectance spectra in the solid state; (b) normalized emission spectra of **2-Pristine** (λ_ex_ 448 nm), **2-Y** (λ_ex_ 430 nm), **2-R** (λ_ex_ 500 nm), and **2-B** (λ_ex_ 615 nm) at 298 K; (c) color under ambient and UV light (λ_ex_ 365 nm), including the black solid (**2-B**), obtained by slow evaporation of a saturated CH_2_Cl_2_ solution of **2** and red (**2-R**) and yellow (**2-Y**) crystals grown by slow diffusion of *n-*hexane into a CH_2_Cl_2_ or CHCl_3_ solution of **2**, respectively; (d) PXRD patterns.

The luminescent properties in the solid state of the different pseudopolymorphs of **2** were also investigated (Figure 9 and Figure S18). Crystals of **2-Y** show an absorption profile (extended to ∼525 nm) and a structured emission band (λ_max_ 540 nm) similar to those of the pristine solid, which are attributed to IL/MLCT transitions originating in the monomer. However, the crystals of the red form **2-R** present a red color due to enhanced absorption in the 500–660 nm region. At 298 K, **2-R** displays a broad and featureless emission at 656 nm, originating from a ^3^MMLCT excited state, in coherence with the short Pt···Pt distances found in the dimer unit of **2-R** crystals. At 77 K, the emission band becomes narrow and the maximum wavelength is red-shifted to 680 nm. As expected, the black form **2-B** exhibits a more red-shifted absorption profile with a maximum at around 700 nm. This form develops at 298 K a narrow unstructured low-energy emission (740 nm), shifted to the near-infrared at low temperature (818 nm), which is typical of the ^3^MMLCT excited state. Based on the black color and the emission color, it is proposed that disordered aggregates with more effective Pt···Pt and π···π interactions, with closer distances, are formed in this amorphous phase (**2-B**), thus leading to a much smaller HOMO–LUMO gap. This black form, **2-B**, displays a slightly shorter lifetime (τ = 12.8 μs) and similar quantum yield (ϕ = 1%) in comparison to the pristine solid (τ = 13.6 μs; ϕ = 1%), while **2-Y** crystals display an increment of the efficiency (ϕ = 5%) with a longer lifetime (τ = 18.7 μs), which may be caused by the order in this crystalline phase. The red microcrystalline solid (**2-R**) displays an important increment in the emission brightness (ϕ = 19%) and a shorter lifetime (6.1 μs), in accordance with the ^3^MMLCT contribution to the emissive excited state. The PXRD patterns found for the pseudopolymorphs **2-Y** and **2-R** show a peak distributions similar to those simulated from the corresponding X-ray diffraction data, although with a different relation of intensities, whereas the phase **2-B** displays broad peaks indicating a lower crystallinity, in coherence with the trouble in obtaining single crystals of this polymorph (Figure 9d).

With the objective of explaining the formation of the pseudopolymorphs of **2**, with different aggregation degrees, the optimization in the gas phase of dimer, trimer and tetramer models with two different arrangements of ligands, head-to-tail (**a**, eclipsed pbt-pic) and head-to-head (**b**, eclipsed pbt-pbt), have been carried out using a dispersion-corrected DFT method at the B3LYP-D3BJ/(6-31G**+LANL2DZ) level of theory. Theoretical calculations of excited states of related dimers, trimers, and tetramers and their dependence on the temperature, employing a periodic QM/MM method with self-consistent charge distribution, have been recently used by Sakaki et al. to explain the emission spectra of crystals of [Pt(CN)_2_(bpy)].^18^

**Figure 10:**
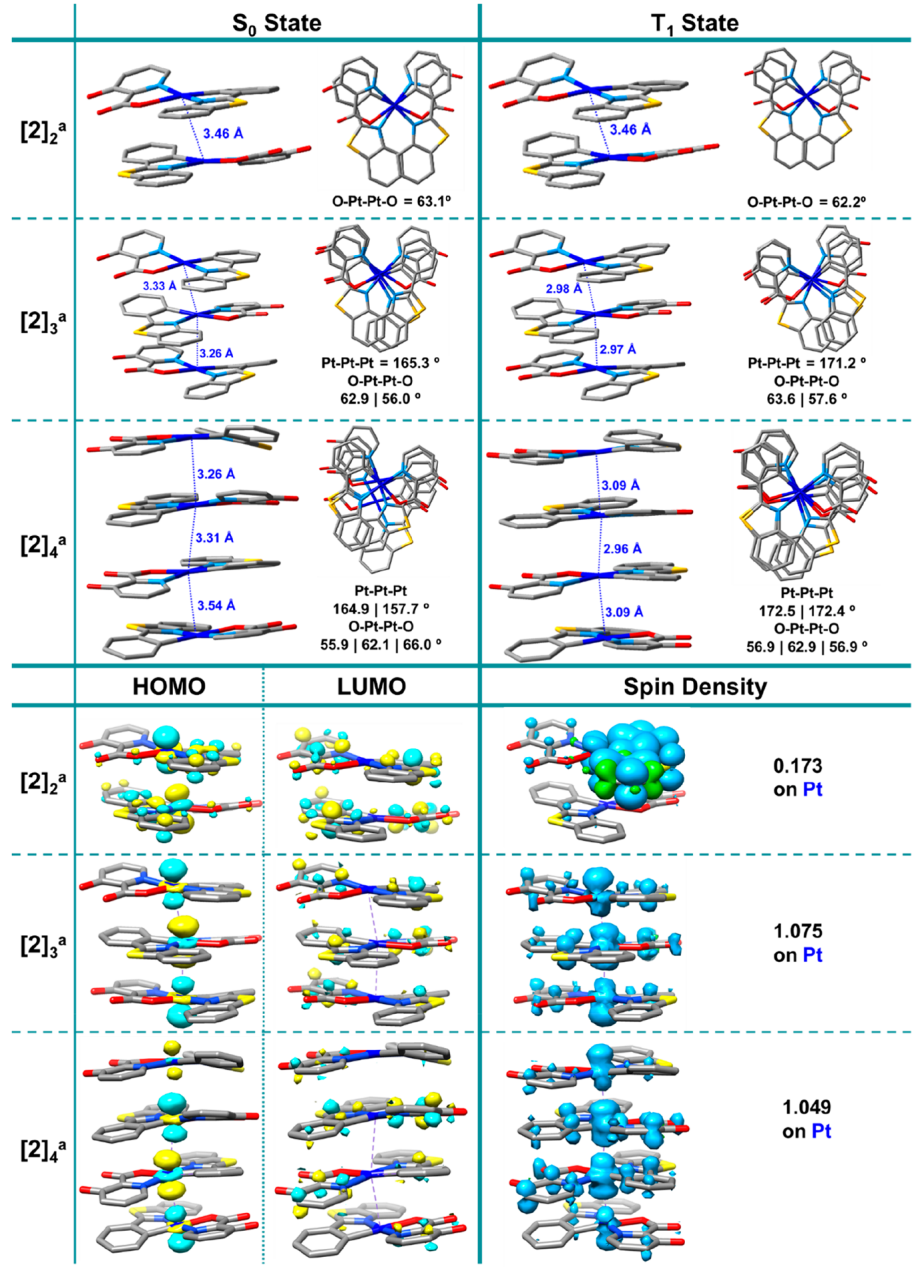
Optimized structures of **[2]**_**2**_^**a**^, **[2]**_**3**_^**a**^, and **[2]**_**4**_^**a**^ models with a head-to-tail disposition at S_0_ and T_1_ states, surface plots of the HOMO and LUMO at the ground state, and spin density at T_1_ (B3LYP-D3BJ/6-31G**).

In our calculations, for all models upon optimization, the platinum units adopt a staggered disposition, thus minimizing steric constraints. The geometries of the calculated structures of **[2]**_**2**_^**a**^, **[2]**_**3**_^**a**^, and **[2]**_**4**_^**a**^ with their Pt···Pt distances, Pt–Pt–Pt and O–Pt–Pt–O angles, the orbitals involved in the electronic transitions (HOMO/LUMO), and the spin density plots are shown in Figure 10. The information for **[2]**_**2**_^**b**^, **[2]**_**3**_^**b**^, and **[2]**_**4**_^**b**^ is included in Figure S19. For the dimer model **[2]**_**2**_^**a**^ (Figure 10) the computed Pt···Pt distance in both S_0_ and T_1_ (3.46 Å) is 0.07 Å longer than that found in the dimer unit of the X-ray structure of **2-R**. Consequently, the density surface is centered in only one of the molecules, entailing an ^3^IL/^3^MLCT nature for the emission. Indeed, the calculated emission energy is blue-shifted in relation to that of the red form at room temperature (644 (**[2]**_**2**_^**a**^) vs 653 nm (**2-R**); Table 3), thus suggesting that emission comes from more extended aggregates. For the trimer **[2]**_**3**_^**a**^ and tetramer **[2]**_**4**_^**a**^ models, the computed Pt···Pt distances are 3.33 and 3.26 Å and 3.26, 3.31, 3.54 Å in S_0_, respectively, being shorter than in the X-ray structure of **2-R** (3.387, 3.726 Å). As in previous theoretical calculations in related systems, the metallophilic distances are shorter in T_1_-optimized geometries (**[2]**_**3**_^**a**^ 2.98, 2.97 Å; **[2]**_**4**_^**a**^ 3.09, 2.96, 3.09 Å) due to the antibonding dσ* nature of the HOMO. The S_1_ transitions of computed **[2]**_**3**_^**a**^ and **[2]**_**4**_^**a**^ were mainly derived from the HOMO → LUMO transitions (∼95%). In both aggregates, the HOMO is located at the Pt atoms (90%), whereas the LUMO is distributed between the pbt cyclometalated ligand (42% (**[2]**_**3**_^**a**^), 38% (**[2]**_**4**_^**a**^)) and the OH-pic coligand (49% (**[2]**_**3**_^**a**^), 52% (**[2]**_**4**_^**a**^)), giving rise to a ^1^MM(L+L′)CT low-energy band, red-shifted in relation to those calculated for the monomer and dimer (S_1_ 550 (**[2]**_**3**_^**a**^), 581 nm (**[2]**_**4**_^**a**^) vs 421 (**2**), 449 nm (**[2]**_**2**_^**a**^); Table 3) and in accordance with the tendency observed in the solid absorption of the red (**2-R**) and black (**2-B)** forms. The computed SOMO-1 is mainly located on the Pt centers (85%), whereas the SOMO is distributed between the pbt (41% (**[2]**_**3**_^**a**^), 36% (**[2]**_**4**_^**a**^)) and the OH-pic (46% (**[2]**_**3**_^**a**^), 50% (**[2]**_**4**_^**a**^)). This result and the corresponding spin density plots in both models (Figure 10 and Figure S19) highlight the ^3^MM(L+L′)CT character of the emission. The calculated emission maxima are red-shifted (789 (**[2]**_**3**_^**a**^) and 883 nm (**[2]**_**4**_^**a**^); Table 3) in relation to those calculated for the monomer and dimer (see Table 3), in line with the experimental data for the yellow (540 nm (**2-Y**)) and red forms (656 nm (**2-R**)), although somewhat overestimated.

**Table 3:** Calculated S_1_ Vertical Excitation Energies and Emission Energies in the Gas Phase for Different Models

model	S_1_ λ(nm)	assignment	Δ*E* emission (T_1_–S_0_)_opt_ (nm)	character
**2**	421	HOMO → LUMO (96%)	653	^3^IL/^3^MLCT
**[2]**_**2**_^a^	449	HOMO → LUMO (96%)	644	^3^IL/^3^MLCT
**[2]**_**3**_^a^	550	HOMO → LUMO (94%)	789	^3^MM(L+L′)CT
**[2]**_**4**_^a^	581	HOMO → LUMO (95%)	883	^3^MM(L+L′)CT
**[2]**_**2**_^b^	498	HOMO → LUMO (99%)	711	^3^MM(L+L′)CT
**[2]**_**3**_^b^	537	HOMO → LUMO (92%)	927	^3^MM(L+L′)CT
**[2]**_**4**_^b^	642	HOMO → LUMO (89%)	1070	^3^MM(L+L′)CT

a Head-to-tail structure.

b Head-to-head structure.

Starting from head-to-head models (**b**), the optimization of the geometries for **[2]**_**2**_^**b**^, **[2]**_**3**_^**b**^, and **[2]**_**4**_^**b**^ leads to staggered structures in the S_0_ with lower O–Pt–Pt–O torsional angles (∼30°, 15°) than those found in the head-to-tail (**a**) models and in **2-R** (∼60–63°). On average, the Pt···Pt distances in S_0_ (3.26 Å (**[2]**_**2**_^**b**^); 3.25, 3.55 Å (**[2]**_**3**_^**b**^); 3.20, 3.20, 3.54 Å (**[2]**_**4**_^**b**^)) and in T_1_ (2.82–3.05 Å) are shorter than those found for the head-to-tail (**a**) models (Figure S19). Consequently, the S_1_ transitions (HOMO → LUMO), with ^1^MM(L+L′)CT character, appear slightly red-shifted (S_1_ 498 **[2]**_**2**_^**b**^), 537 (**[2]**_**3**_^**b**^), 642 (**[2]**_**4**_^**b**^) Table 3) in relation to (**a**) models. The calculated energy emission from the corresponding T_1_ excited states (^3^MM(L+L′)CT in nature; see spin density plots and the SOMO and SOMO-1 orbitals in Table S10) are notably red-shifted in relation to the head-to-tail (**a**) models (927 vs 789 nm (**[2]**_**3**_) and 1070 vs 883 nm (**[2]**_**4**_); Table 3). In short, the calculated red-shifted absorptions and emissions induced by stacking follow the order **[2]**_**4**_ > **[2]**_**3**_ > **[2]**_**2**_, the calculated energies being red-shifted in the head-to-head (**b**) models in relation to the head-to-tail models (**a**).

Taking into account these calculations and the experimental absorption and emission data of the black form **2-B**, we tentatively propose that this polymorph could adopt a head-to-head (**b**) arrangement of the Pt units with closer Pt···Pt separations and reduced HOMO–LUMO gaps. In support of this suggestion, the following features are highlighted. First, the closest calculated value to the experimental absorption band of **2-B** (603 nm with a tail to 700 nm; Figure 9a) is found for **[2]**_**4**_^**b**^ in a head-to-head model (642 nm). In addition, although the calculated emission in **[2]**_**4**_^**b**^ is red-shifted in relation to the experimental emission (calculated 1070 nm vs 740 nm, 298 K; 818 nm, 77 K), it should be noted that these calculations usually overestimate the values of the observed emissions. Finally, as noted above, the black **2-B** phase reverts to the unsolvated yellow phase **2-Y** after 5 h in the oven at 100 °C. As crystals of the **2-Y** phase display a slipped head-to-head orientation, this structural transformation seems to be more likely to start from an initial head-to-head disposition of the molecules.

**Figure 11:**
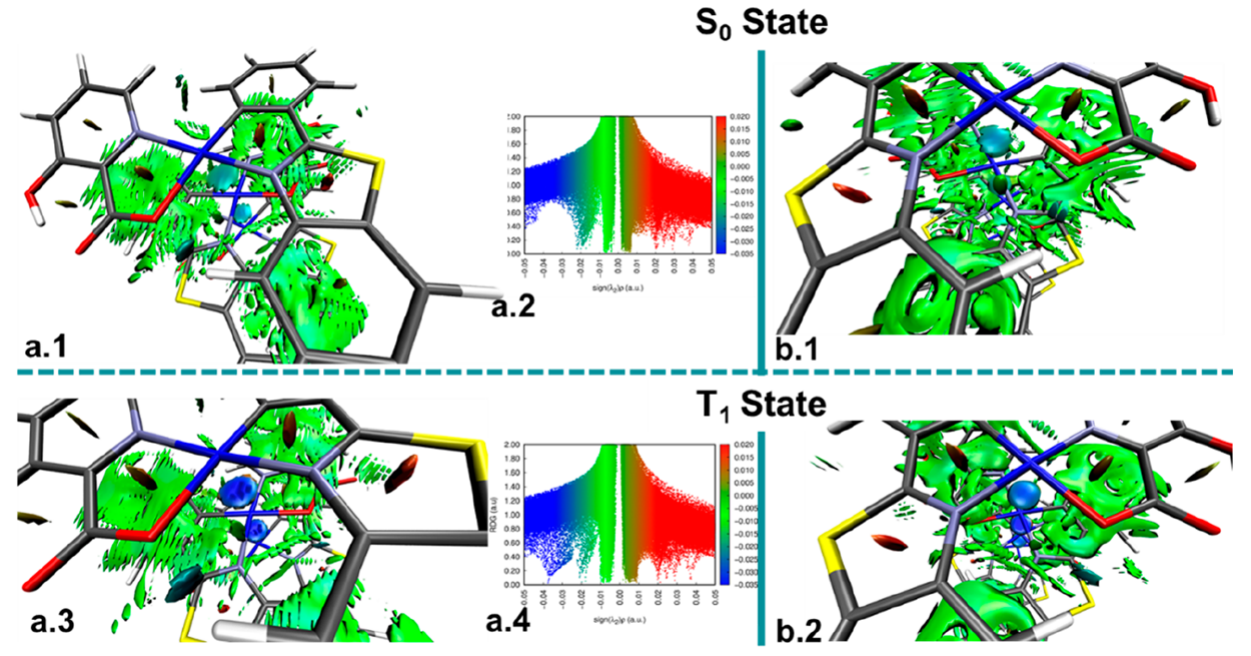
NCI analysis of (a) **[2]**_**3**_^**a**^ and (b) **[2]**_**4**_^**a**^ (head-to-tail) S_0_ and T_1_ optimized structures at the B3LYP-D3BJ level (isovalue 0.3 au).

Finally, the intermolecular interactions in the trimer **[2]**_**3**_ and tetramer **[2]**_**4**_ models have been also analyzed by carrying out a study of the noncovalent interactions (NCIs) on the optimized geometries (B3LYP-D3BJ) in the S_0_ and T_1_ states. Isosurfaces and 2D plots of RDG vs sign(λ_2_)ρ are summarized in Figure 11 and Figures S20 and S21. As can be observed in all models, large green isosurface regions are observed in accordance with the extensive π···π stacking among chelates of the different molecules. S_0_ optimized states display a green-blue region between the Pt atoms, assigned to weak Pt···Pt interactions, which become stronger in the T_1_ optimized models, appearing as an intense dark blue disk between the Pt centers. This strengthening of the metallophilic interactions can be observed also in the RDG vs sign(λ_2_)ρ plots (Figures 11a2,a4), in which new peaks in the negative attractive region (blue ∼−0.04) of the 2D representation appear, indicating a shortening of the Pt···Pt distances in the T_1_ state. Thus, this NCI study supports the ^3^MM(L+L′)CT character of the emission.

## Conclusions

In summary, we report the synthesis, structures, optical properties, and theoretical calculations of two new cycloplatinated(II) complexes based on pbt and picolinate ligands. We have been able to isolate three pseudopolymorphs with different aggregation degrees for complex **2**: yellow **2-Y**, orange-red **2-R** (**2·0.5CH**_**2**_**Cl**_**2**_), and black **2-B** (**2·0.75CH**_**2**_**Cl**_**2**_). This highlights that the richness of the polymorphism in **2**, in contrast to **1**, lies in the involvement of the hydroxyl moiety in intermolecular donor–acceptor H-bonding interactions with the CH_2_Cl_2_ solvent. **2-R** and **2-B** can be transformed to **2-Pristine**. A comparative study of their crystal packing revels a head-to-head stairlike packing for **1** and **2-Y** with π···π stacking interactions among the chelate rings and long Pt···Pt distances (5.634 (**1**), 5.426 Å (**2-Y**)), whereas **2-R** exhibits a head-to-tail columnar disposition with alternating short and long Pt···Pt distances (∼3.4; ∼3.7 Å) together with π···π contacts, highlighted by an NCI theoretical study. These results show that the orientation of the neighboring Pt monomer chromophores is sensitive to the ratio between the CH_2_Cl_2_ and the Pt molecules and that furthermore modulate the degree of Pt···Pt and/or π···π interactions, showing different colors and optical properties. As expected, a higher aggregation degree, associated with Pt···Pt and/or π···π contacts, results in a darker color and lower emission energy. Pristine samples of **1** and **2** display outstanding reversible mechanochromic behavior involving a contrast color emission change from yellow to orange-red, which arises from a phase transition between a crystalline state and an amorphous state. Theoretical calculations using dimer/trimer and tetramer models with head-to-tail (**a**) and head-to-head (**b**) disposition suggest that the ^3^MM(L+L′)CT excited states are most likely generated in trimers and tetramers. More relevant Pt···Pt interactions, especially in their T_1_ states, are associated with a change from mixed ^3^IL/^3^MLCT character in the monomer to mixed ^3^MM(L+L′)CT in the oligomers. From this study, we tentatively propose a head-to-head oligomer arrangement for the black phase **2-B**, which shows emission in the near-infrared range. This phase increases the limited family of compounds that emit in the NIR.

## Experimental Section

### General Comments

All reactions were carried out under an atmosphere of dry argon, using standard Schlenk techniques. Solvents were obtained from a solvent purification system (M-BRAUN MS SPS-800). Elemental analyses were carried out with a PerkinElmer CHNS/O 2400 Series II microanalyzer. Mass spectra were recorded on a Microflex MALDI-TOF Bruker (MALDI) spectrometer operating in the linear and reflector modes using dithranol as the matrix. IR spectra of powders were obtained on a PerkinElmer Spectrum UATR Two FT-IR spectrophotometer, with the diamond crystal ATR accesory, covering the region between 4000 and 450 cm^–1^; data processing was carried out with Omnic. NMR spectra were recorded on a Bruker AVANCE ARX 400 spectrometer at 298 K. Chemical shifts are reported in parts per million (ppm) relative to external standards (SiMe_4_ for ^1^H and ^13^C{^1^H}), and all coupling constants are given in hertz (Hz). The UV–vis absorption spectra were measured with a Hewlett-Packard 8453 spectrophotometer. Diffuse reflectance UV–vis (DRUV) spectra were carried out in SiO_2_ pellets, using a Shimazdu UV-3600 spectrophotometer with a Harrick Praying Mantis accessory, and recalculated following the Kubelka–Munk function. Excitation and emission spectra were obtained with a Edimburg FLS 1000 spectrofluorimeter. Lifetime measurements were performed with an Edinburgh FLS 1000 spectrofluorimeter with μF2 pulse lamp (power, 100 W; fuse, 3.15 amp A/S). The absolute quantum yields were determined with a Hamamatsu Absolute PL Quantum Yield Measurement System. The powder X-ray diffraction (XRD) patterns were obtained at room temperature by using a Rigaku Miniflex II instrument with graphite-monochromated Cu Kα radiation operating at 30 kV and 15 mA. PXDR patterns were collected between 2θ values of 3 and 60° with a 2θ step angle of 0.03° and an angle dwell of 1 s. The complex [Pt(pbt)Cl(DMSO)]^15d^ was prepared according to the published procedure. Other commercially available reagents were used as received.

### Synthesis of [Pt(pbt)(pic-κ*-N,O*)] (**1**)

2-Picolinic acid (0.024 g, 0.192 mmol) and excess Na_2_CO_3_ (∼0.25 g) were added to a suspension of [Pt(pbt)Cl(DMSO)] (0.103 g, 0.192 mmol) in 20 mL of acetone. After 5 h of stirring at room temperature the suspension was evaporated to dryness and the residue treated with CH_2_Cl_2_ (25 mL) and deionized water (40 mL). The organic phase was extracted, dried with anhydrous MgSO_4_, and filtered through Celite. The filtrate was dried and the residue treated with *n*-hexane (10 mL) to give **1** as a yellow solid (0.071 g, 70%). Anal. Calcd for C_19_H_12_N_2_O_2_PtS (527.046): C, 43.27; H, 2.29; N, 5.31; S, 6.08. Found: C, 43.52; H, 2.59; N, 5.63; S, 6.13. MALDI-TOF (+): *m*/*z* (%) 526.92 [M]^+^ (100). IR (ATR) (cm^–1^): ν(C=O) 1660 (s), ν(C–O) 1336 (s). ^1^H NMR (400 MHz, CDCl_3_): δ 9.30 (d, ^3^*J*_H–H_ = 8, H^7^), 9.26 (d, ^3^*J*_H–H_ = 8, ^3^*J*_Pt–H_ = 45, H^6’^), 8.26 (dd, ^3^*J*_H–H_ = 8, ^4^*J*_H–H_ = 1, H^3′^), 8.18 (td, ^3^*J*_H–H_ = 8, ^4^*J*_H–H_ = 1, H^4′^), 7.83 (d, ^3^*J*_H–H_ = 8, H^4^), 7.68 (t, ^3^*J*_H–H_ = 8, ^4^*J*_H–H_ = 1, H^6^), 7.66 (t, ^3^*J*_H–H_ = 8, ^4^*J*_H–H_ = 1, H^5′^), 7.59 (dd, ^3^*J*_H–H_ = 7.4, ^4^*J*_H–H_ = 1, H^11^), 7.51 (d, ^3^*J*_H–H_ = 7.4, H^8^), 7.48 (td, ^3^*J*_H–H_ = 8, ^4^*J*_H–H_ = 1, H^5^), 7.24–7.19 (m, 2H, H^9^, H^10^). ^13^C{^1^H} NMR (100.6 MHz, CDCl_3_): δ 181.6 (s, C^2^), 172.9 (s, C = O), 152.6 (s, C^2′^), 150.1 (s, C^7a^), 149.3 (s, C^6′^), 142.7 (s, C^3a^), 142.1 (s, C^12^), 139.2 (s, C^4′^), 136.5 (s, C^13^), 131.7 (s, C^8^), 130.8 (s, C^9^), 128.6 (s, C^6^), 127.9 (s, C^3′^), 127.8 (s, C^2’^), 126.0 (s, C^5^), 125.7 (s, C^11^), 124.2 (s, C^10^), 122.2 (s, C^7^), 121.9 (s, C^4^).

### Synthesis of [Pt(pbt)(OH-pic-κ*-N,O*)] (**2**)

Complex **2** was obtained as a yellow solid (0.100 g, 79%) following the same procedure as **1**, starting from 3-hydroxy-2-picolinic acid (0.034 g, 0.233 mmol) and [Pt(pbt)Cl(DMSO)] (0.121 g, 0.233 mmol). Anal. Calcd for C_19_H_13_N_3_O_2_PtS (542.046): C, 41.99; H, 2.23; N, 5.15; S, 5.90. Found: C, 42.09; H, 2.54; N, 5.43; S, 6.19. MALDI-TOF (+): *m*/*z* (%) 542.82 [M]^+^ (100). IR (ATR) (cm^–1^): ν(O–H) 2695 (s), ν(C=O) 1641 (s), ν(C–O) 1326 (s). ^1^H NMR (400 MHz, CDCl_3_): δ 13.19 (s, −OH), 9.14 (d, ^3^*J*_H–H_ = 7.5, H^7^), 8.74 (d, ^3^*J*_H–H_ = 5, ^3^*J*_Pt–H_ = 46, H^6’^), 7.82 (d, ^3^*J*_H–H_ = 8, H^4^), 7.68 (d, ^3^*J*_H–H_ = 8, H^8^), 7.63 (t, ^3^*J*_H–H_ = 8, H^6^), 7.54–7.45 (m, 4H, H^5,11,5′,4′^), 7.22 (td, ^3^*J*_H–H_ = 8, ^4^*J*_H–H_ = 1.5, H^9^), 7.17 (t, ^3^*J*_H–H_ = 7.5, H^10^).

### X-ray Diffraction

X-ray intensity data were collected using molybdenum graphite monochromated (Mo Kα) radiation with a Nonius-κCCD diffractometer at a temperature of 173 K with an Oxford Cryosystem temperature controller using the DENZO and SCALEPACK suite programs for **1** or with a Bruker APEX-II diffractometer at a temperature of 100 K using APEX-II programs for **2**. Structures were solved by intrinsic phasing using SHELXT^19^ with the WinGX graphical user interface.^20^ Multiscan absorption corrections were applied to all the data sets and refined by full-matrix least squares on *F*^2^.^19^ Hydrogen atoms were positioned geometrically, with isotropic parameters *U*_iso_ = 1.2*U*_eq_ (parent atom) for aromatic hydrogens and CH_2_ and *U*_iso_ = 1.5*U*_eq_ (parent atom) for methyl groups. Finally, the structures showed some residual peaks in the vicinity of the platinum atoms but had no chemical meaning.

### Theoretical Calculations

Calculations were carried out with the Gaussian 16 package^21^ using the Becke three-parameter functional combined with the Lee–Yang–Parr correlation functional (B3LYP)^22^ with the Becke–Johnson D3BJ correction.^23^ Optimizations on the singlet state (S_0_) were performed using as a starting point the molecular geometry obtained through an X-ray diffraction analysis. No imaginary frequency was found in the vibrational frequency analysis of the final equilibrium geometries. The basis set used was the LanL2DZ effective core potential for Pt and 6-31G(d,p) for the ligand atoms.^24^ DFT and TD-DFT calculations were carried out in solution using the polarized continuum model approach^25^ (PCM) implemented in the Gaussian 16 software, in the presence of CH_2_Cl_2_ and in the gas phase. The predicted emission wavelengths were obtained by the energy difference between the triplet state at its optimized geometry and the singlet state at the triplet geometry. The results were visualized with GaussView 6, and orbitals and spin density surfaces plots were created with Chimera 1.16.^26^ Overlap populations between molecular fragments were calculated using the GaussSum 3.0 software.^27^

### Noncovalent Interactions

Noncovalent interaction isosurfaces were obtained with Multiwfn (v 3.7).^28^ The results were visualized with the Visual Molecular Dynamics (VMD) program (v 19.3)^29^ for 3D plots and with gnuplot (v 5.2.8) for 2D representations.^30^

## Supporting Information Available

Characterization of complexes (NMR spectra, crystal data), photophysical properties, and computational details (PDF)
