# Supplementary material for: Polymorphism and Mechanochromism in 2-Phenylbenzothiazole Cyclometalated PtII Complexes with Chelating N∧O Ligands
Source: Inorg Chem. 2022 Nov 28;61(49):20043–56. doi: 10.1021/acs.inorgchem.2c03423 (PMC9749027; doi:10.1021/acs.inorgchem.2c03423)
Supplement: Supplementary file 1 — ic2c03423_si_001.pdf [file ic2c03423_si_001.pdf]

Supporting Information

Polymorphism and Mechanochromism in 2-Phenylbenzothiazole Cyclometalated Pt^{II} Complexes with Chelating N^O Ligands.

David Gómez de Segura, Elena Lalinde and M. Teresa Moreno**

Departamento de Química-Centro de Síntesis Química de La Rioja, (CISQ), Universidad de La Rioja, 26006, Logroño, Spain. E-mail: elena.lalinde@unirioja.es; teresa.moreno@unirioja.es

Contents: Page

1.- NMR Spectra.. S2
2.- Crystal Structures.. S5
3.- Photophysical Properties and Theoretical calculations.............................. S9

1.- NMR spectra

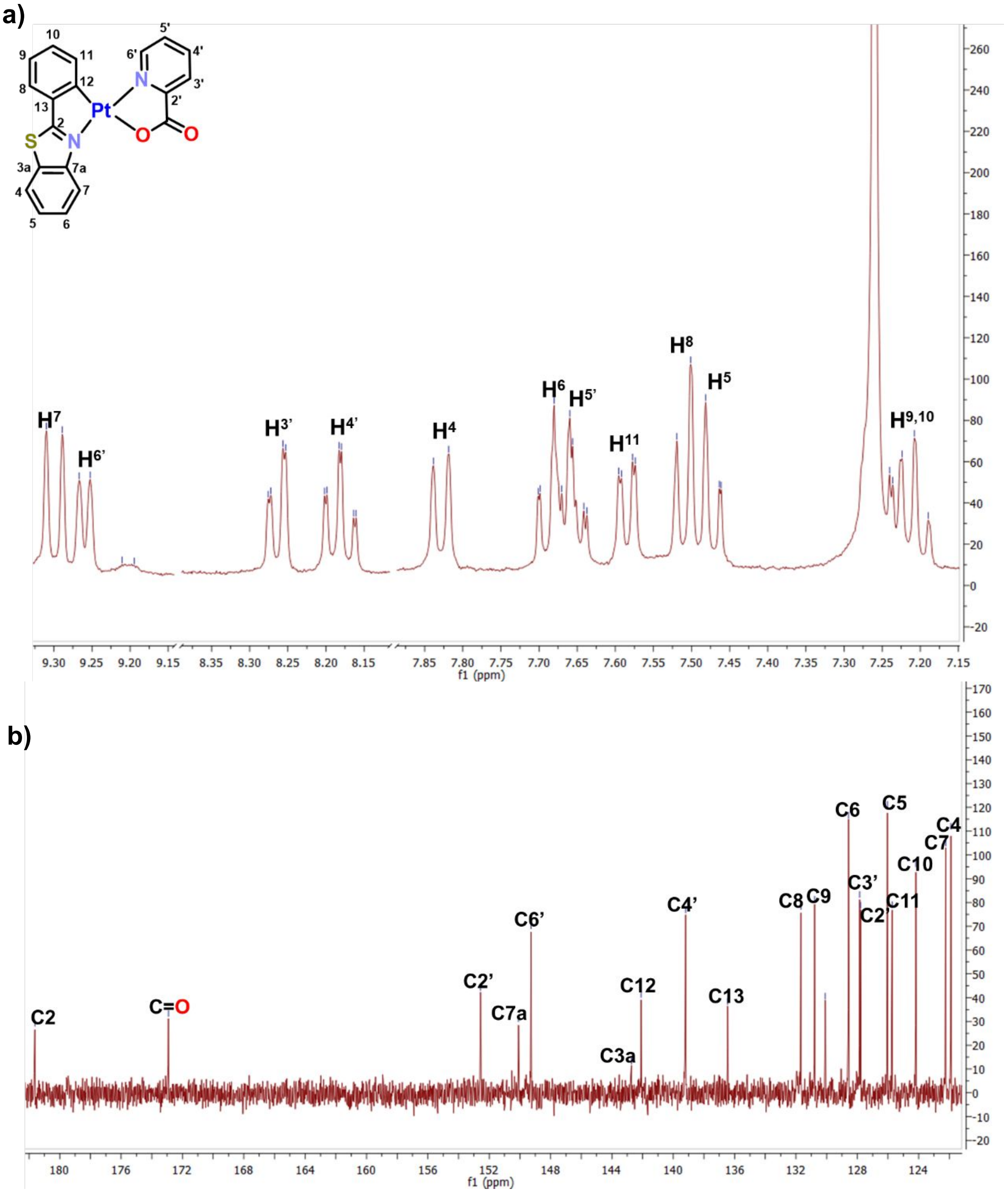

Figure S1. NMR spectra of **1** in $CDCl_3$ at 298 K (a) ^{1}H, (b) $^{13}C\{^{1}H\}$

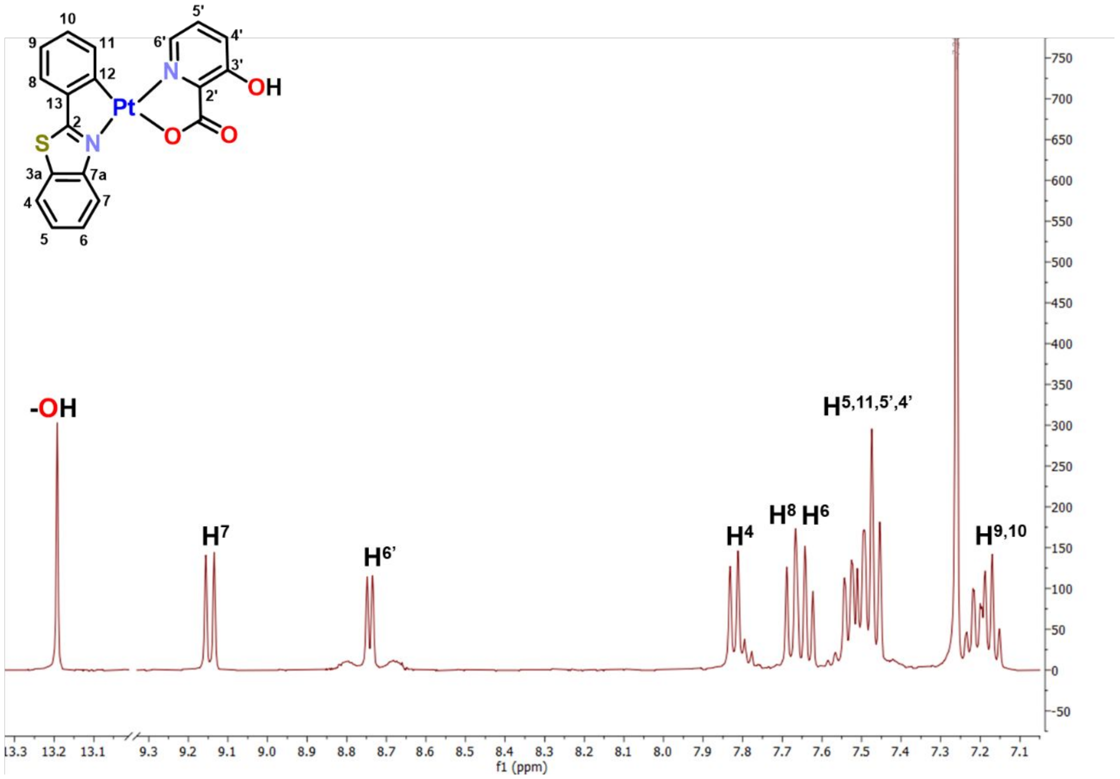

Figure S2. ^{1}H NMR spectra of **2** in $CDCl_3$ at 298 K.

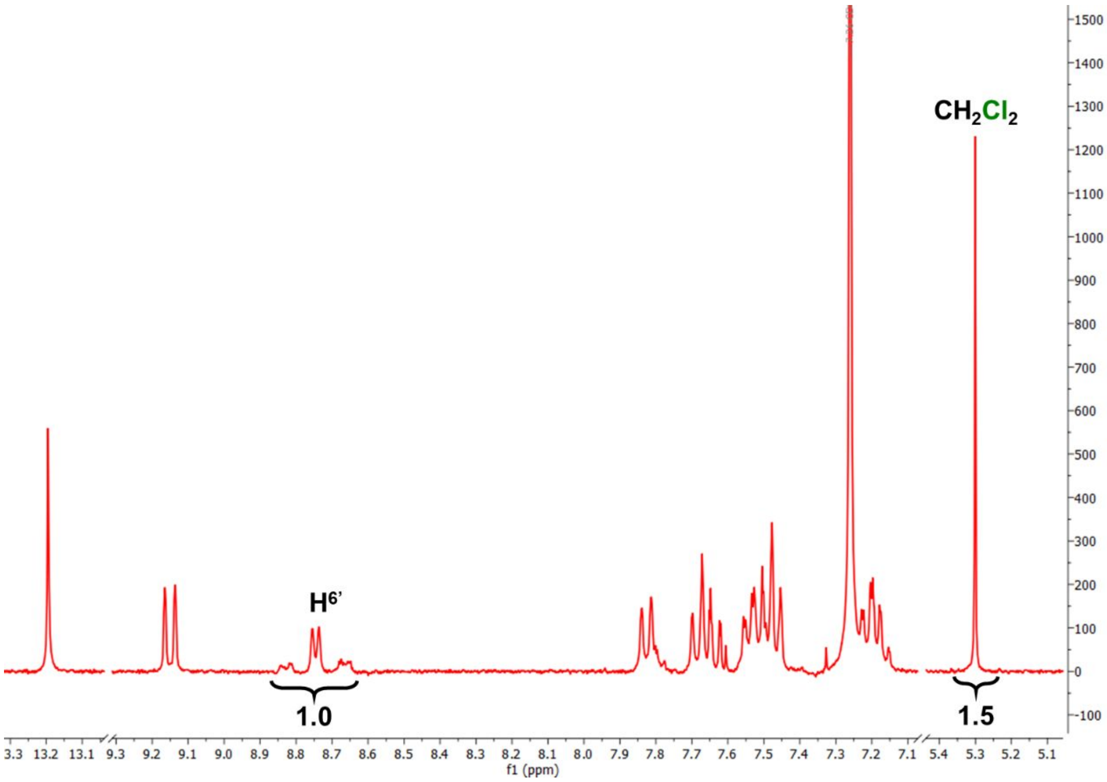

Figure S3. ^{1}H NMR spectra of **2-B** in $CDCl_3$ at 298 K.

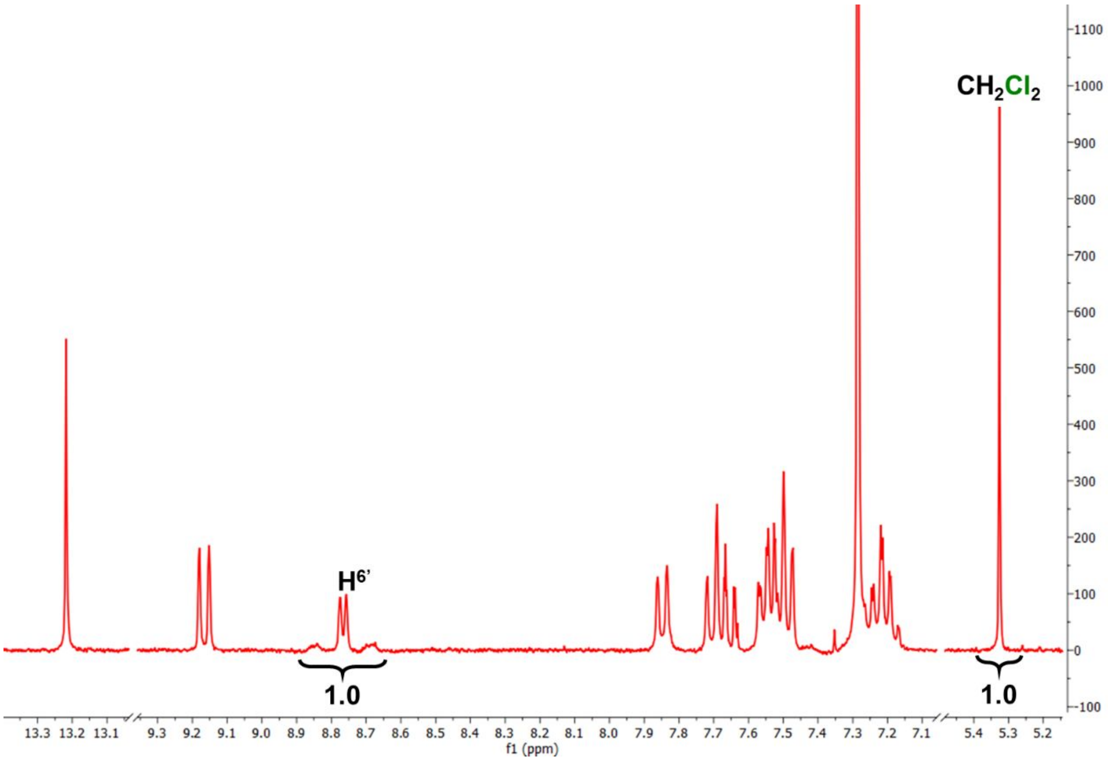

Figure S4. ^{1}H NMR spectra of **2-R** in $CDCl_3$ at 298 K.

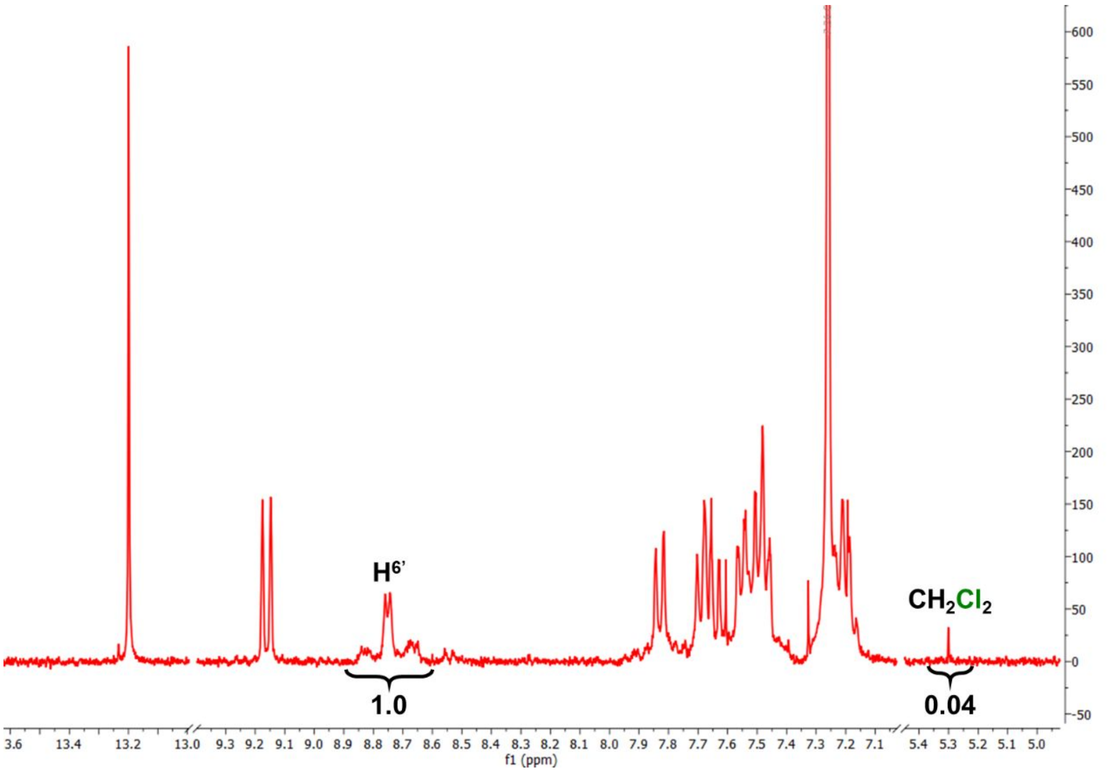

Figure S5. ^{1}H NMR spectra of **2-Y/2-Pristine** in $CDCl_3$ at 298 K.

2.- Crystal Structures

Table S1. X-ray Crystallographic data for **1**, **2-Y** and **2·0.5CH$_2$Cl$_2$** (**2-R**)

	**1**	**2-Y**	**2·0.5CH$_2$Cl$_2$ (2-R)**
**Empirical formula**	$C_{19}H_{12}N_2O_2PtS$	$C_{19}H_{12}N_2O_3PtS$	$C_{19}H_{12}N_2O_3PtS$
**Molecular weight**	527.46	543.46	543.46
**T (K)**	173(1)	298(2)	100(2)
**Wavelength (Å)**	0.71073	0.71076	0.71076
**Crystal system**	Orthorombic	Orthorhombic	Monoclinic
**Space group**	$Pna2_1$	$P2_12_12_1$	$P2_1$
**Crystal size (mm)**	0.200 x 0.150 x 0.100	0.399 x 0.040 x 0.020	0.152 x 0.136 x 0.042
**a (Å)**	22.3835(10)	5.4258(2)	6.9465(3)
**b (Å)**	12.0942(4)	13.1513(7)	16.4759(6)
**c (Å)**	5.6344(2)	22.6938(12)	30.4656(13)
**α (°)**	90.0	90.0	90.0
**β (°)**	90.0	90.0	91.0
**γ (°)**	90.0	90.0	90.0
**V (Å^3)**	1525.29(10)	1619.35(14)	3486.3(2)
**Z**	4	4	8
**Density (calculated) (Mg/cm^3)**	2.297	2.229	2.233
**Absorption coefficient (mm^{-1})**	9.352	8.817	8.348
**F(000)**	1000	1032	2232
**θ range for data collection (°)**	3.369 to 26.727	3.098 to 26.733	2.356 to 28.134
**Index ranges**	-7<=h<=7, -15<=k<=15, -29<=l<=29	-6<=h<=6, -16<=k<=16, -28<=l<=28	-9<=h<=9, -21<=k<=20, -40<=l<=40
**Reflections collected**	1778	84851	182081
**Independent reflections**	1778 [R(int) = 0]	3415 [R(int) = 0.0397]	16463 [R(int) = 0.0326]
**Data / restraints / parameters**	1778 / 1 / 227	3415 / 0 / 239	16463 / 1 / 1068
**Goodness-of-fit on F2**	1.031	1.078	1.352
**Final R indices [I>2σ(I)]**[a]	R1 = 0.0215, wR2 = 0.0509	R1 = 0.0115, wR2 = 0.0255	R1 = 0.0188, wR2 = 0.0446
**R indices (all data)**[a]	R1 = 0.0269, wR2 = 0.0528	R1 = 0.0122, wR2 = 0.0259	R1 = 0.0198, wR2 = 0.0450
**Largest diff. peak and hole (e Å^{-3}) (dmin/dmax)**	0.747 and -1.229	0.368 and -0.554	2.302 and -1.224

[a] $R1 = \Sigma(|F_o| - |F_c|)/\Sigma|F_o|$; $wR2 = [\Sigma w(F_o^2 - F_c^2)^2/\Sigma wF_o^2]^{1/2}$; goodness of fit = $\{\Sigma[w(F_o^2 - F_c^2)^2]/(N_{obs} - N_{param})\}^{1/2}$; $w = [\sigma^2(F_o) + (g_1P)^2 + g_2P]^{-1}$; $P = [\max(F_o^2;0 + 2F_c^2]/3$.

Table S2. Selected distances (Å) and angles (°) for complexes **1**, **2-Y** and **2·0.5CH$_2$Cl$_2$** (**2-R**)

**1**			
**Distances (Å)**		**Angles (°)**	
Pt(1)-N(1)	2.010(6)	N(1)-Pt(1)-O(1)	96.3(2)
Pt(1)-N(2)	2.042(6)	N(2)-Pt(1)-O(1)	79.0(2)
Pt(1)-C(1)	2.020(8)	C(18)-N(2)-Pt(1)	113.9(4)
Pt(1)-O(1)	2.118(6)	N(2)-C(18)-C(19)	115.7(6)
C(6)-C(7)	1.467(9)	O(1)-C(19)-C(18)	114.2(6)
		C(19)-O(1)-Pt(1)	115.9(5)
		N(1)-Pt(1)-C(1)	81.0(3)
		C(1)-Pt(1)-N(2)	103.8(2)
**2-Y**			
**Distances (Å)**		**Angles (°)**	
Pt(1)-N(1)	2.028(3)	N(1)-Pt(1)-O(1)	96.86(11)
Pt(1)-N(2)	2.042(3)	N(2)-Pt(1)-O(1)	79.54(12)
Pt(1)-C(1)	2.007(3)	C(18)-N(2)-Pt(1)	113.2(2)
Pt(1)-O(1)	2.113(3)	N(2)-C(18)-C(19)	115.9(3)
C(6)-C(7)	1.442(5)	O(1)-C(19)-C(18)	116.6(4)
		C(19)-O(1)-Pt(1)	113.9(2)
		N(1)-Pt(1)-C(1)	80.51(13)
		C(1)-Pt(1)-N(2)	103.20(14)
**2·0.5CH$_2$Cl$_2$ (2-R)**			
**Distances (Å)**		**Angles (°)**	
Pt(1)-N(1)	2.011(5)	N(1)-Pt(1)-O(1)	96.7(2)
Pt(1)-N(2)	2.021(5)	N(2)-Pt(1)-O(1)	81.0(2)
Pt(1)-C(1)	2.002(6)	C(18)-N(2)-Pt(1)	113.4(4)
Pt(1)-O(1)	2.129(4)	N(2)-C(18)-C(19)	116.2(5)
C(6)-C(7)	1.451(9)	O(1)-C(19)-C(18)	117.8(5)
		C(19)-O(1)-Pt(1)	111.1(4)
		N(1)-Pt(1)-C(1)	80.8(2)
		C(1)-Pt(1)-N(2)	101.7(2)

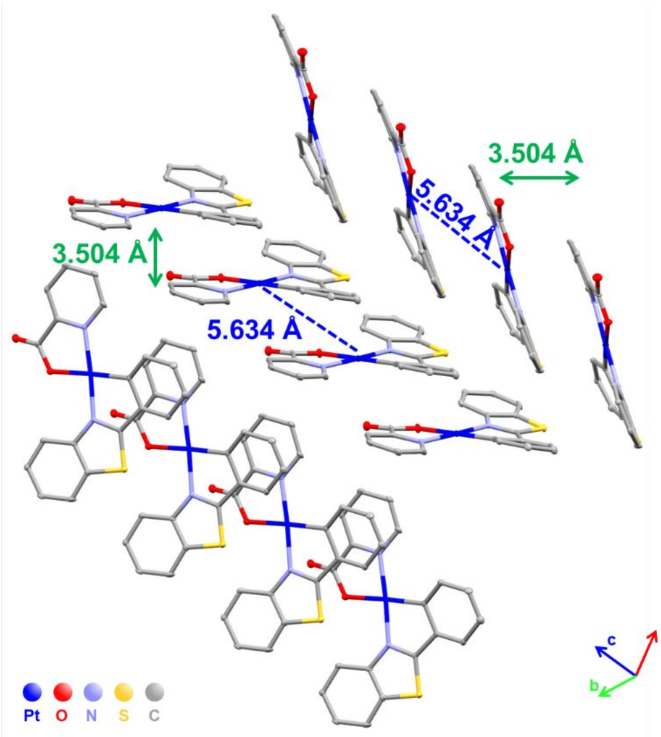

Figure S6. Disposition of the columns in the crystal packing of **1**.

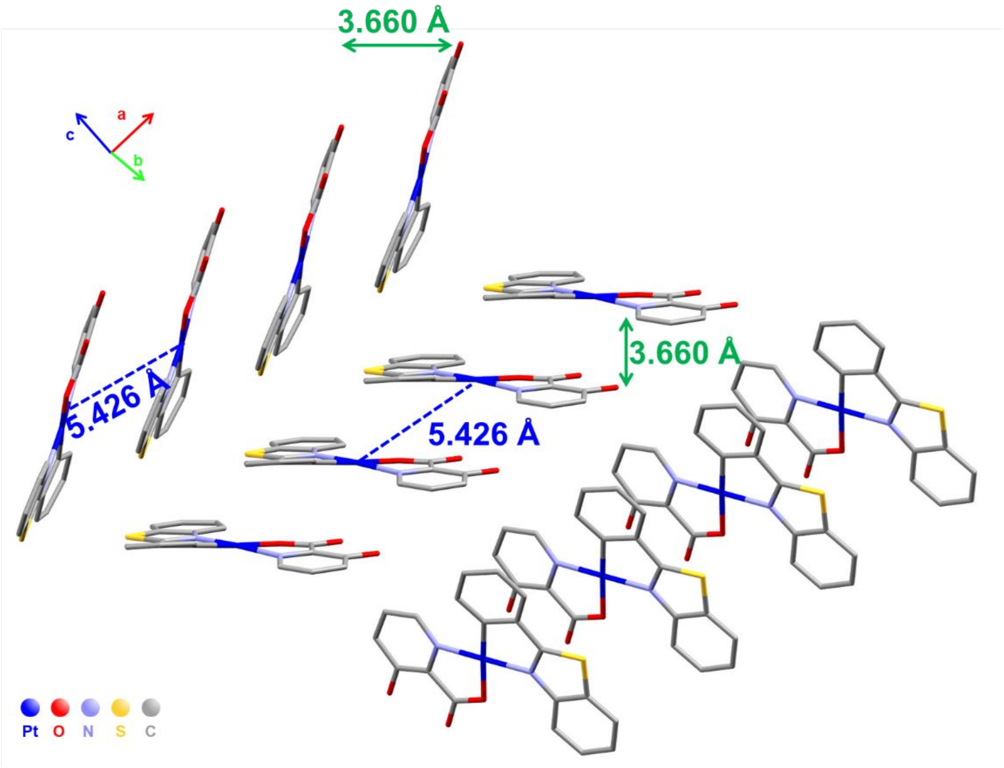

Figure S7. Disposition of the columns in the crystal packing of **2-Y**.

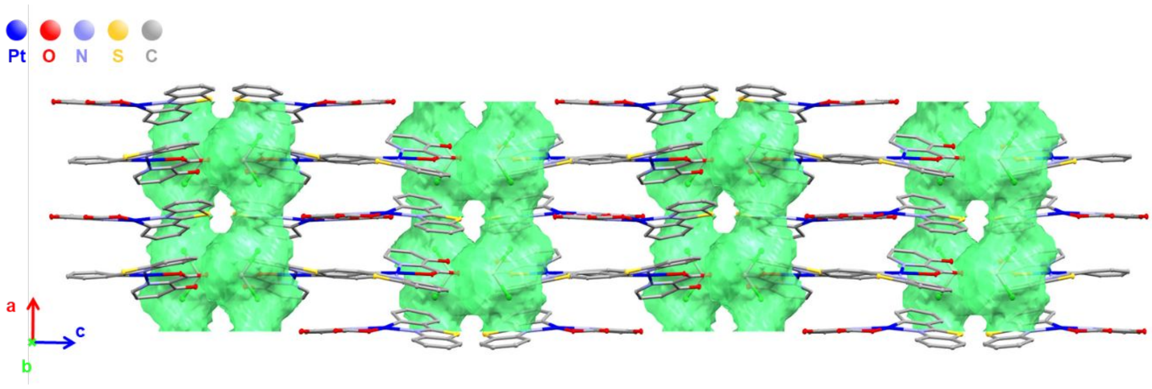

Figure S8. Packing structure of **2-R** with the solvent channels marked in green along *b* axis.

3.- Photophysical Properties and Theoretical Calculations

Table S3. Absorption data for complexes **1 – 2** (CH_2Cl_2 5 × 10^{-5} M and solid)

Compound	Media	λ_{abs}/nm (ε × 10^{-3}/$M^{-1}cm^{-1}$)
**[Pt(pbt)(pic-*κ*-*N,O*)] (1)**	**CH_2Cl_2**	320 (14.32), 335 (13.76), 353 (8.02), 370 (9.61), 407 (2.95), 431 (2.41)
	**1-Pristine**	314, 342, 374, 391, 421, 442, 498_{sh}, 535_{sh}
	**1-Ground**	317, 394, 433, 496, 502_{sh}, 550 tail to 620
	**1-Ground + CH_2Cl_2**	327, 387, 418, 440, 502_{sh}, 534
**[Pt(pbt)(OH-pic-*κ*-*N,O*)] (2)**	**CH_2Cl_2**	321 (16.60), 335 (13.60), 355 (8.27), 371 (10.03), 408 (2.93), 429 (2.26)
	**2-Pristine**	333, 391, 431, 501_{sh}
	**2-Ground**	333, 388, 435, 497_{sh}, 537_{sh}, 597_{sh} tail to 638
	**2-Ground + $CHCl_3$**	341, 391, 434, 491_{sh}, 524
	**2-Y**	341, 391, 434, 492_{sh}, 525
	**2-R**	329, 381, 437, 517_{sh}, 547_{sh}, 615 tail to 660
	**2-B**	348, 387, 440, 566, 603 tail to 700

Table S4. Selected vertical excitations energies singlets (S_n) and first triplets (T_n) computed by TD-DFT/SCRF (CH_2Cl_2) with the orbitals involved

	State	λ/nm	*f*	Transition (% Contribución)
**1**	T_1	507.9	-	HOMO→LUMO (68%), HOMO→L+1 (10%)
	T_2	418.4	-	H-1→LUMO (60%), HOMO→LUMO (21%)
	T_3	391.9	-	HOMO→L+1 (65%)
	S_1	419.6	0.0591	HOMO→LUMO (97%)
	S_2	369.2	0.0293	H-2→LUMO (39%), H-1→LUMO (18%), HOMO→L+1 (39%)
	S_3	367.4	0.0284	H-2→LUMO (26%), H-1→LUMO (15%), HOMO→L+1 (56%)
	S_4	346.7	0.2076	H-2→LUMO (31%), H-1→LUMO (60%)
	S_6	321.1	0.2019	H-4→LUMO (18%), H-3→LUMO (66%)
	S_7	318.9	0.0564	H-4→LUMO (43%), H-4→L+1 (21%), H-3→LUMO (22%)
**2**	T_1	509.2	-	HOMO→LUMO (67%), HOMO→L+1 (15%)
	T_2	416.2	-	H-1→LUMO (63%), HOMO→LUMO (17%)
	T_3	406.2	-	H-4→LUMO (31%), H-4→L+1 (20%), H-3→LUMO (10%)
	S_1	418.3	0.0583	HOMO→LUMO (96%)
	S_2	370.3	0.0161	H-2→LUMO (65%), H-1→LUMO (22%), HOMO→L+1 (10%)
	S_3	367.9	0.0475	HOMO→L+1 (83%)
	S_4	349.4	0.2253	H-2→LUMO (25%), H-1→LUMO (68%)
	S_6	322.3	0.2526	H-3→LUMO (90%)
	S_7	314.3	0.0631	H-2→L+1 (15%), H-1→L+1 (67%)

Table S5. Composition (%) of Frontier MOs in terms of ligands and metals in the ground state in CH_2Cl_2.

**1**				
**Orbital**	**Energy (eV)**	**Pt**	**pbt**	**pic**
**LUMO+5**	-0.29	17	80	3
**LUMO+4**	-0.44	3	96	1
**LUMO+3**	-0.56	48	36	16
**LUMO+2**	-1.22	2	2	97
**LUMO+1**	-1.81	3	34	63
**LUMO**	-2.19	5	63	32
**HOMO**	-5.89	34	58	8
**HOMO-1**	-6.34	41	50	9
**HOMO-2**	-6.46	84	13	3
**HOMO-3**	-6.62	14	83	3
**HOMO-4**	-6.96	7	8	85
**HOMO-5**	-7.11	40	46	15

2				
**Orbital**	**Energy (eV)**	**Pt**	**pbt**	**OH-pic**
**LUMO+5**	-0.32	17	80	3
**LUMO+4**	-0.46	4	95	2
**LUMO+3**	-0.55	39	33	28
**LUMO+2**	-0.82	10	8	82
**LUMO+1**	-1.88	3	41	56
**LUMO**	-2.26	5	56	39
**HOMO**	-5.96	32	63	5
**HOMO-1**	-6.39	40	50	10
**HOMO-2**	-6.49	85	9	6
**HOMO-3**	-6.65	13	82	6
**HOMO-4**	-6.85	4	13	83
**HOMO-5**	-7.16	38	42	20

a)

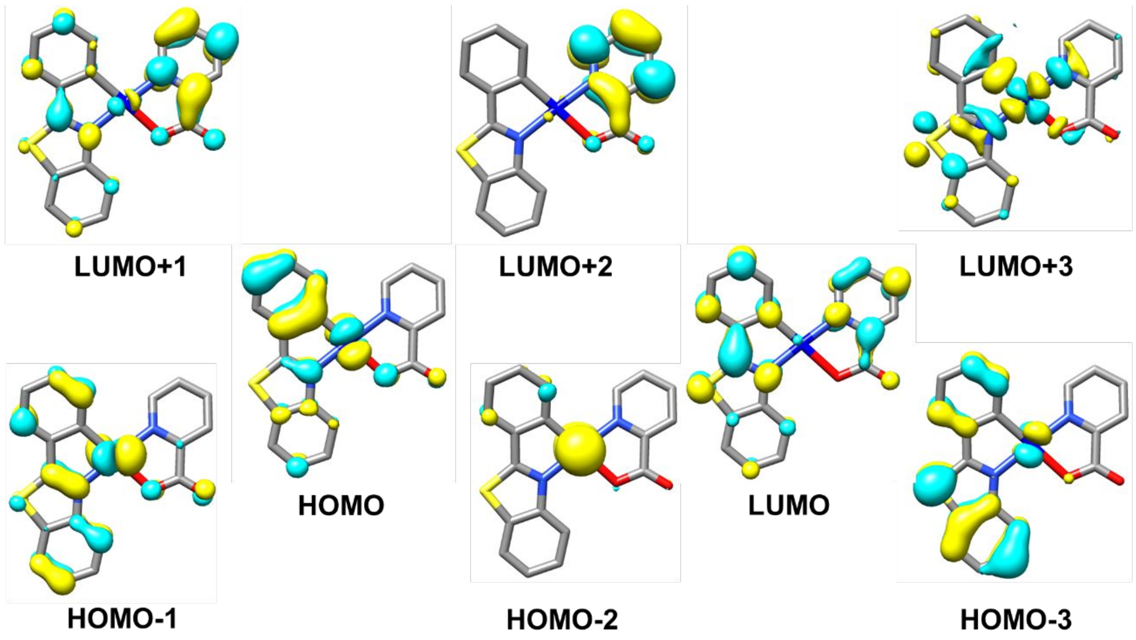

b)

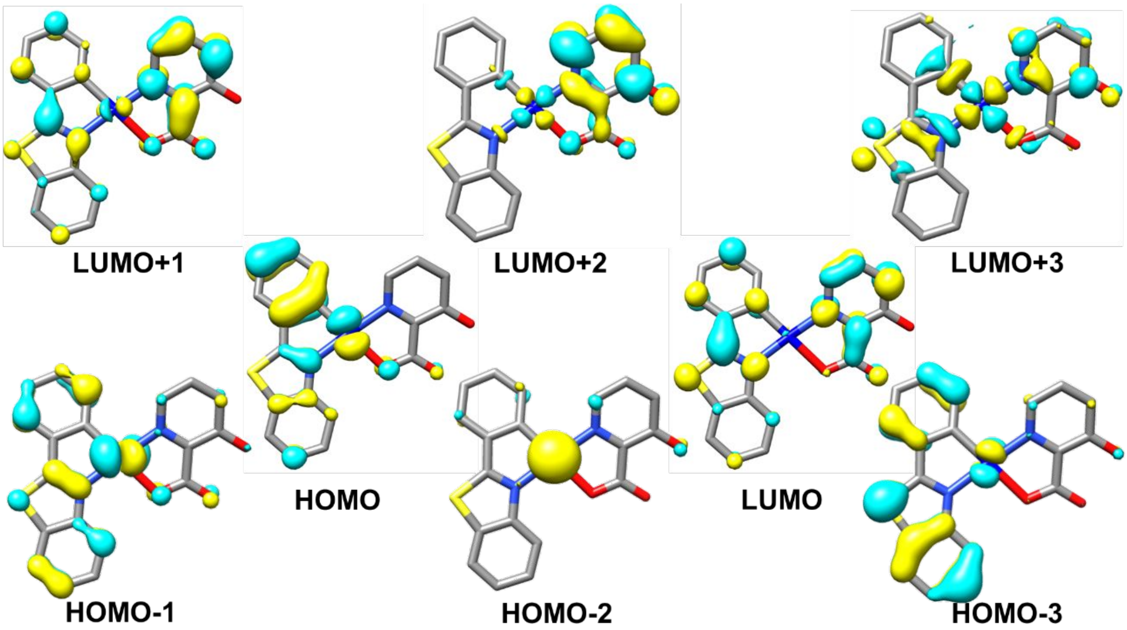

Figure S9. Selected frontier Molecular Orbitals for a) **1** and b) **2** in the ground state in solution (CH_2Cl_2).

Table S6. Selected parameter of DFT optimized geometries for ground state and triplet state in CH_2Cl_2 (PCM model)

**1**			
**Parameter**	**X-Ray**	**S_0**	**T_1**
Pt(1) - C(1)	2.020(8)	2.02098	1.99768
Pt(1) - N(1)	2.010(6)	2.06117	2.01859
Pt(1) - N(2)	2.042(6)	2.06833	2.07525
Pt(1) - O(1)	2.118(6)	2.15721	2.15635
C(1)-Pt(1)-N(1)	81.0(3)	79.93944	81.44960
N(1)-Pt(1)-O(1)	96.3(2)	98.60018	97.78380
O(1)-Pt(1)-N(2)	79.0(2)	78.34029	78.33138
N(2)-Pt(1)-C(1)	103.8(2)	103.62464	103.00736
**2**			
**Parameter**	**X-Ray**	**S_0**	**T_1**
Pt(1) - C(1)	2.002(6)	2.01132	1.99002
Pt(1) - N(1)	2.024(2)	2.05241	2.01025
Pt(1) - N(2)	2.039(2)	2.06366	2.07013
Pt(1) - O(1)	2.118(2)	2.17595	2.17628
C(1)-Pt(1)-N(1)	80.50(11)	80.02927	81.43326
N(1)-Pt(1)-O(1)	97.25(9)	99.14010	98.44990
O(1)-Pt(1)-N(2)	79.50(9)	78.54697	78.47883
N(2)-Pt(1)-C(1)	102.91(11)	102.92907	102.29053

Table S7. DFT optimized geometries for ground state and triplet state (in CH_2Cl_2)

**1**	
**Singlet (S_0)**	**Triplet (T_1)**
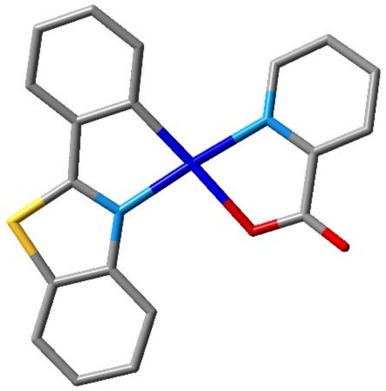	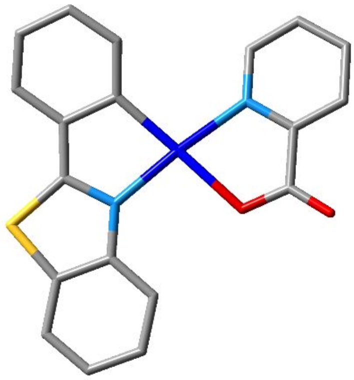
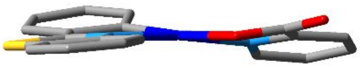	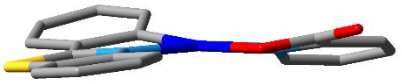

**2**	
**Singlet (S_0)**	**Triplet (T_1)**
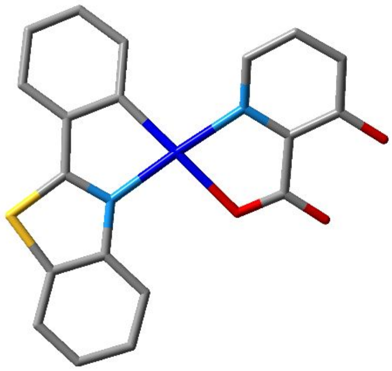	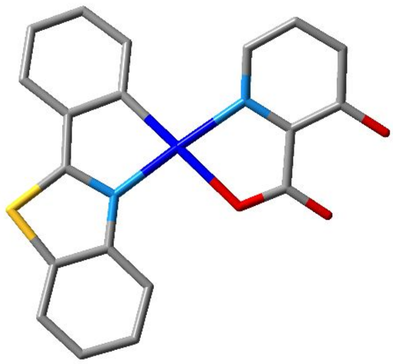
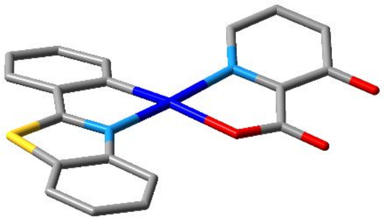	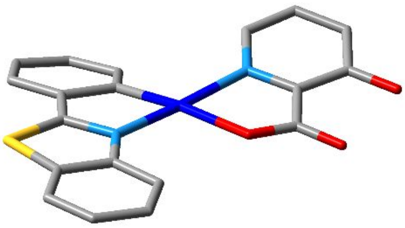

Table S8. Photophysical data in solution and in polymeric film (PS).

Complex	Media	[M]	T[a] (K)	λ_{em} / nm (λ_{ex} / nm)	τ (μs)	ϕ
**1**	PS	10%	298	546, 590, 638, 706_{sh} (438)	4.8[a]	0.05
		5%	298	546, 587, 640, 714_{sh} (435)	6.7[a]	0.09
		1%	298	546, 588, 636, 708_{sh} (434)	7.3[a]	0.17
	CH_2Cl_2	1×10^{-3}	298	541, 583, 631 (440)	13.2[b]	0.07[b,c] 0.03[b,d]
			77	700 (470); 725 (510), 750 (540); 775 (560); 800 (580)		
		5×10^{-4}	298	541, 583, 631 (431)		
			77	542, 592, 680, 708, 740 (420); 742, 764, 800 (536)		
		5×10^{-5}	298	541, 583, 633 (430)		
			77	544, 592, 680, 730 (438); 730 (507)		
	THF	5×10^{-4}	298	540, 585, 630 (440)	12.8[b]	0.14[b,c] 0.03[b,d]
			77	545, 590, 650, 680 (460)		
		5×10^{-5}	298	540, 585, 630 (440)		
			77	545, 590, 650, 680 (446)		
**2**	PS	10%	298	542, 589, 640, 720 (440)	3.9[a] [546] 7.4[a] [720]	0.06
		5%	298	545, 588, 640, 720_{sh} (440)	6.1[a]	0.11
		1%	298	547, 586, 631 (440)	6.8[a]	0.15
	CH_2Cl_2	5×10^{-4}	298	542, 584, 628 (420)	13.4[b]	0.07[b,c] 0.02[b,d]
			77	544, 593, 665,757 (437); 767 (540)		
		5×10^{-5}	298	542, 584, 628 (415)		
			77	542, 587, 645 (420)		
	THF	5×10^{-5}	298	540, 582, 630 (410)	12.6[b]	0.11[b,c] 0.03[b,d]
			77	537, 581, 630 (415)		

[a] Average value of two components. [b] 5×10^{-5} M and 298 K. [c] Degassed solution [d] Oxygenated.

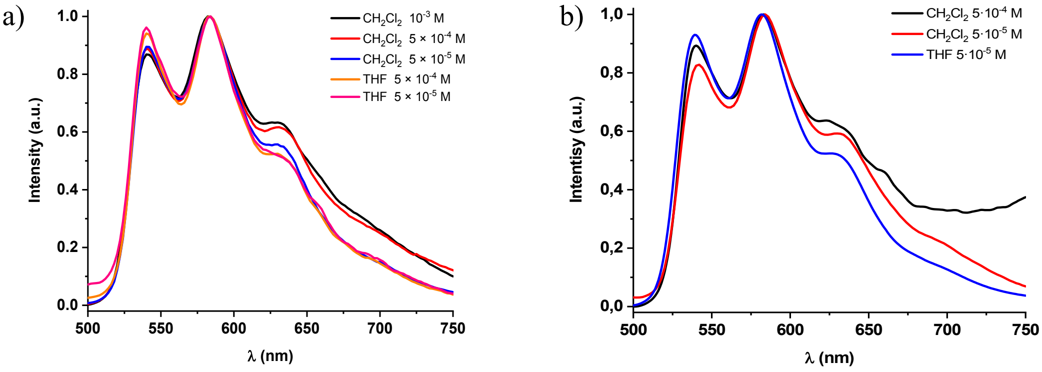

Figure S10. Emission of a) **1** in CH_2Cl_2 5 × 10^{-5} to 10^{-3} M and THF 5 × 10^{-5} to 5 × 10^{-4} M and b) **2** in CH_2Cl_2 5 × 10^{-5} to 5 × 10^{-4} M and THF 5 × 10^{-5} M (λ_{exc} 430-440 nm) at 298 K

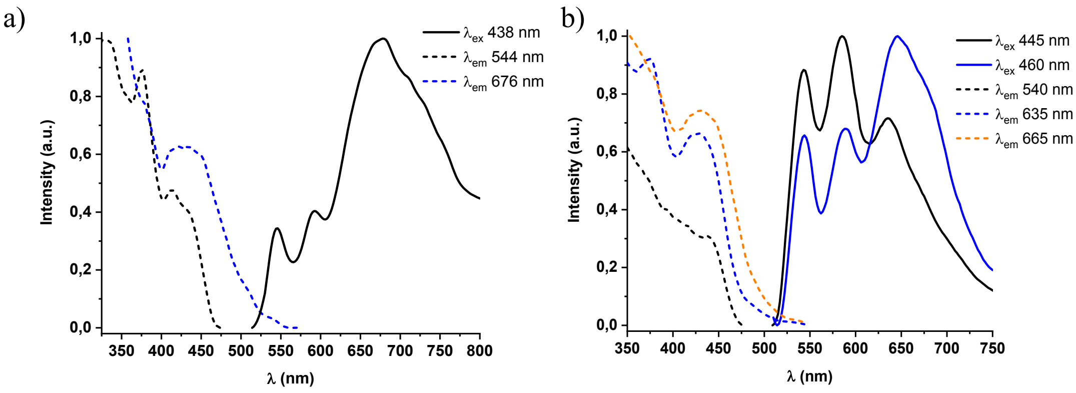

Figure S11. Excitation (····) and Emission (□) of **1** in CH_2Cl_2 (a) and THF (b) 5 × 10^{-5} M at 77 K

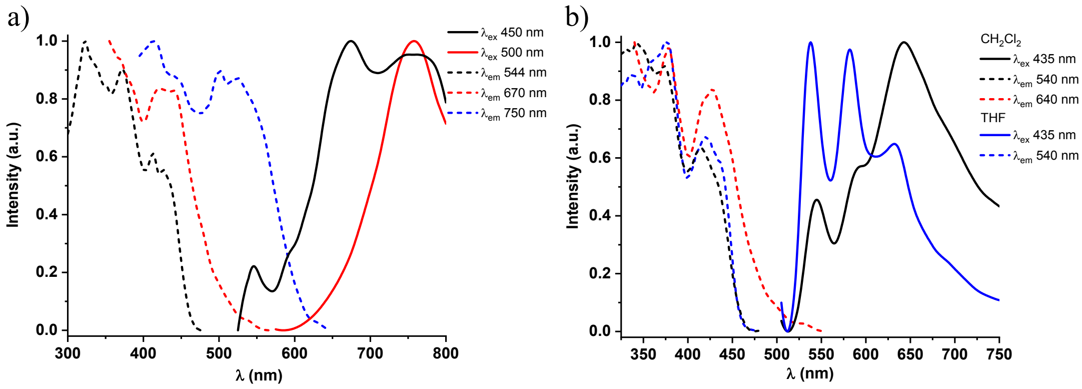

Figure S12. Excitation (····) and Emission (□) of **2** in a) CH_2Cl_2 5 × 10^{-4} M and b) CH_2Cl_2 and THF 5 × 10^{-5} M at 77 K

Table S9. Plots and composition (%) of the frontier MOs of the first triplet state in CH_2Cl_2

**1**	
**SOMO**	**SOMO-1**
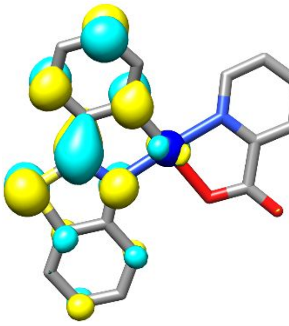	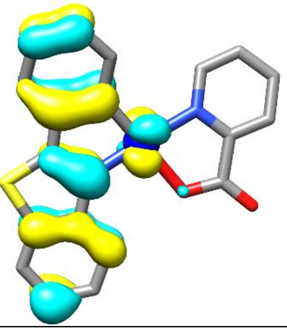
pbt 92%, pic 3%, Pt 5%	pbt 83%, pic 2%, Pt 15%
**2**	
**SOMO**	**SOMO-1**
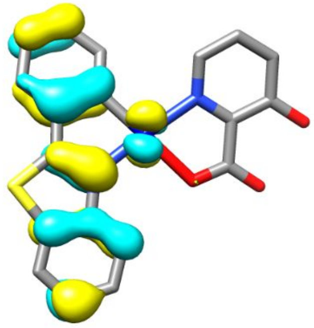	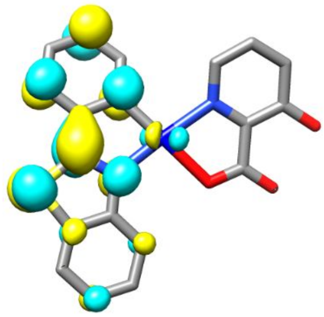
pbt 92%, OH-pic 3%, Pt 5%	pbt 84%, OH-pic 1%, Pt 15%

Mechanochromic behavior of complex 1

Upon mechanical grinding in an agate mortar, this complex changes its color from yellow to orange and the luminescence varies under UV light from the structured yellow emission at 543 nm to a broad red emission without vibronic structure at 700 nm (**Figure S13**), whose maxima is slightly shifted at 77 K (725 nm, λ_{exc} 536 nm, **Figure S14**), attributed to a ^{3}MMLCT excited state. As is typical of this excited state, the emission maximum is shifted from 685 to 712 nm (298 K) and from 670 to 730 nm (77 K) depending on the wavelength used (**Figure S15**). The emission quantum yields of these forms range from ϕ 1% in **1-Pristine** to 3% in **1-Ground**, with lifetimes of 12.5 μs (**1-Pristine**) to 8.5 μs (**1-Ground**). As expected, the yellow **1-Pristine** solid shows a broad absorption band up to 440 nm (with shoulders at 498 and 535 nm), whereas **1-Ground** phase shows an absorption spectrum extending to 620 nm (**Figure S13b**), most likely due to ^{1}MMLCT transitions based on Pt···Pt interactions, in coherence with its color. The powder X-ray diffraction (PXRD) patterns show that **1-Pristine** is crystalline in nature, being the PXRD peaks simulated from the cif files of the structure of single crystals of **1** (**Figure S13d**), suggesting that the solid has a molecular packing similar to that of the crystals of **1**. Upon grinding, the PXRD pattern becomes broad and weak, indicating that the crystalline solid is transformed into amorphous aggregates. Upon treating the ground solid powder **1-Ground** with a few drops of CH_2Cl_2, the orange color turns back to yellow quickly, and both, the absorption and emission spectra are similar to those obtained for **1-Pristine**. In coherence, the intense and narrow peaks are partially recovered in the PXRD pattern. As expected in **Figure S13e**, the reversible process of the mechano-induced luminescence of **1** has been repeated for 6 cycles without distinct chemical degradation.

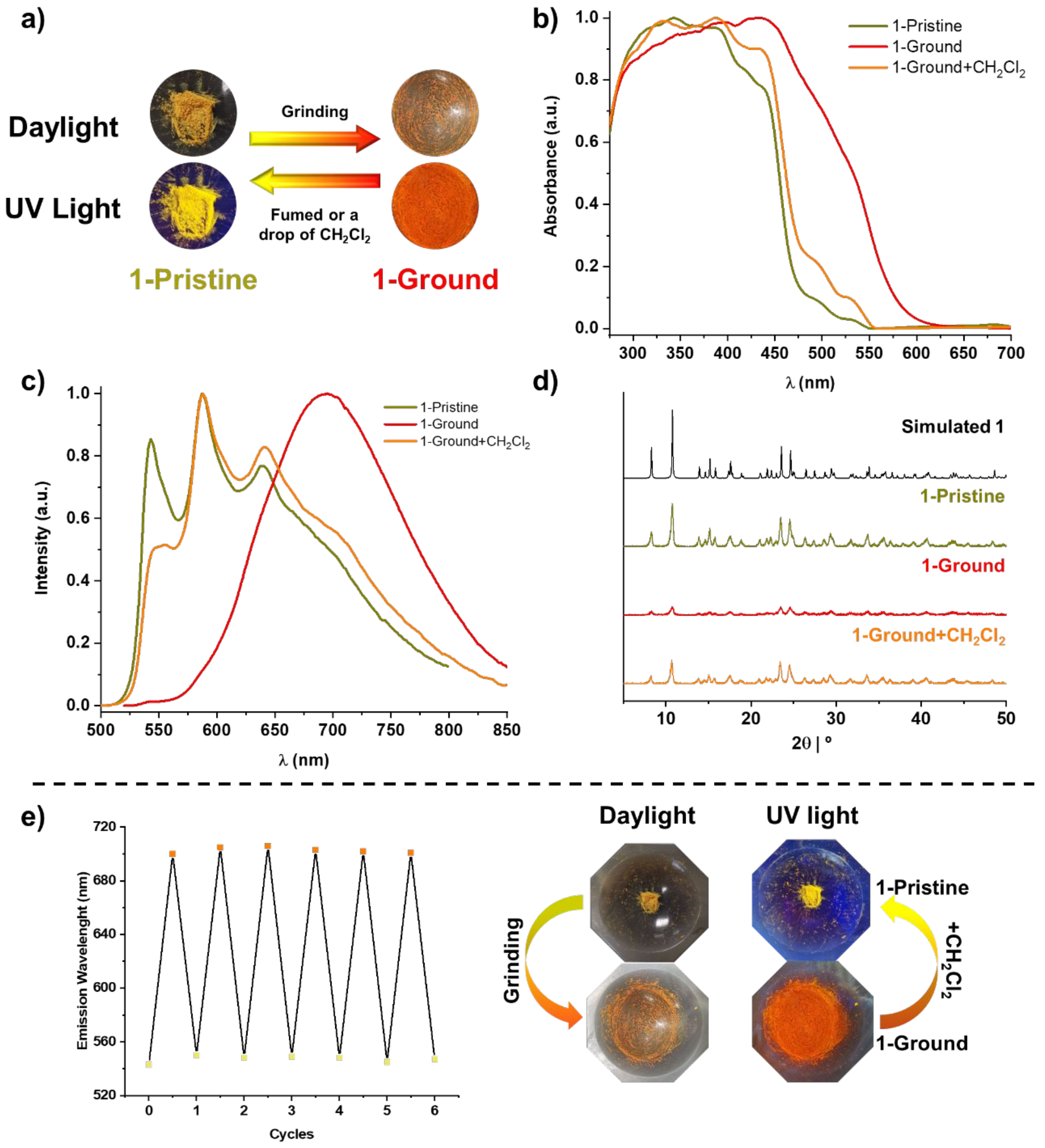

Figure S13. Reversible mechanochromism of **1**: a) Color changes of **1** by grinding and after the addition of one drop of CH_2Cl_2 to the ground solid (Daylight and UV light (λ_{ex} 365 nm). b) Normalized absorption spectra calculated from their reflectance spectra in solid state. c) Normalized emission spectra of **1-Pristine** powder (λ_{ex} 443 nm), after grinding (λ_{ex} 500 nm) and after the addition of one drop of CH_2Cl_2 (λ_{ex} 420 nm) at 298 K. d) Changes in the PXRD patterns by mechanical grinding and addition of CH_2Cl_2 for **1**. e) Plot of emission wavelength changes of **1** during 6-cycles of being exposed to pressure and then to CH_2Cl_2.

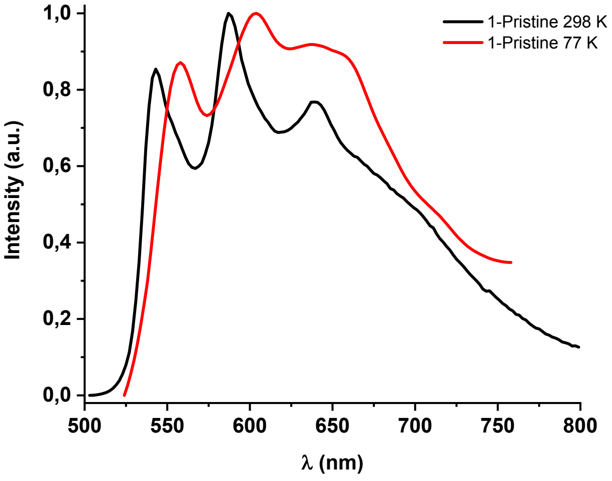

Figure S14. Emission of **1-Pristine** at 298 K (λ_{ex} 445 nm) and 77 K (λ_{ex} 430 nm)

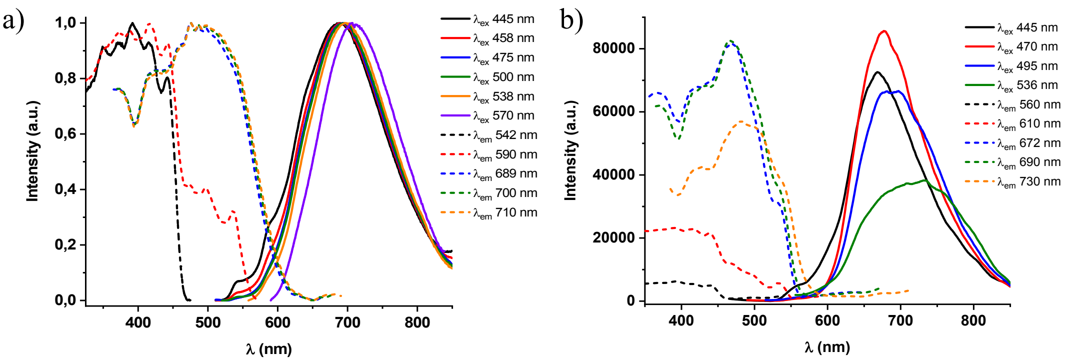

Figure S15. Excitation (····) and Emission (□) of **1-Ground** a) at 298 K and b) 77 K

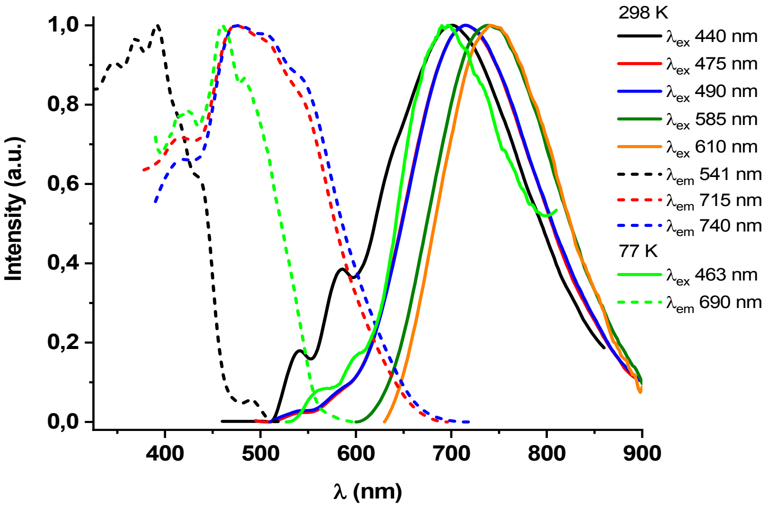

Figure S16. Excitation (····) and Emission (□) of **2-Ground** at 298 K and 77 K.

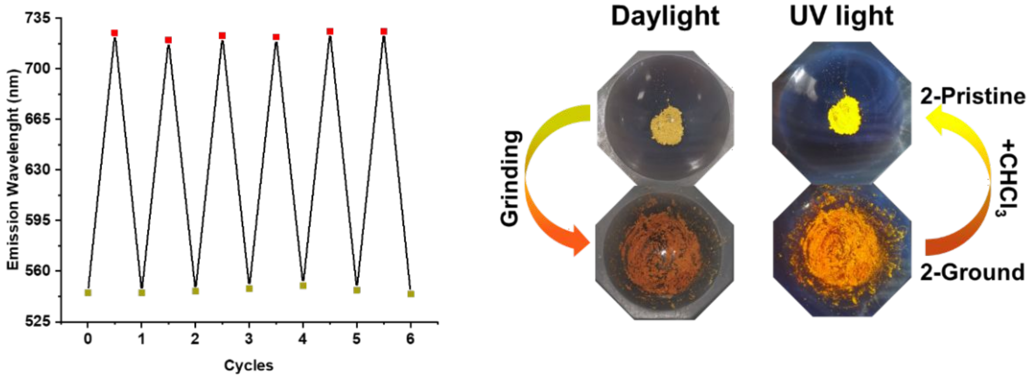

Figure S17. Plot of emission wavelength changes of **2** during 6-cycles of being exposed to pressure and then to $CHCl_3$.

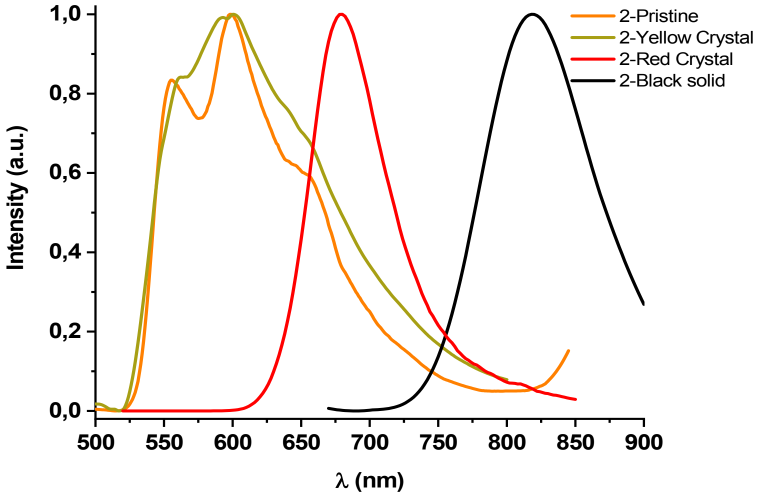

Figure S18. Emission of **2-Pristine** (λ_{ex} 440 nm), **2-Y-crystal** (λ_{ex} 420 nm), **2-R-crystal** (λ_{ex} 500 nm) and **2-B** (λ_{ex} 550 nm) at 77 K.

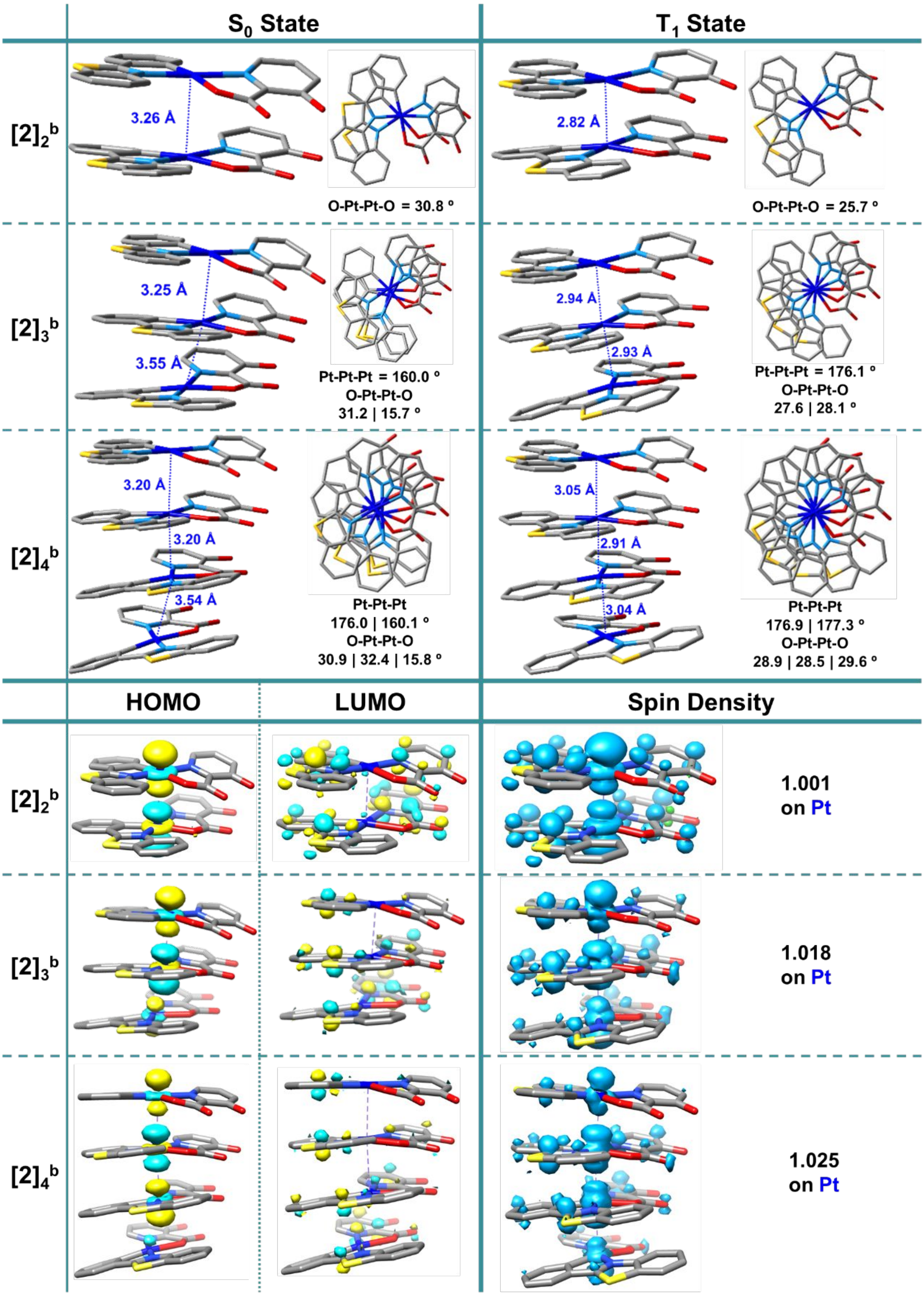

Figure S19. Optimized structures of $\mathbf{[2]_2^b}$, $\mathbf{[2]_3^b}$ and $\mathbf{[2]_4^b}$ models with a head-to-head disposition at S_0 and T_1 states. Surface plots of HOMO and LUMO at ground state and spin density at T_1 [B3LYP-D3BJ/6-31G**]

Table S10. Plots and composition (%) of the frontier MOs and spin density of the first triplet state in gas phase

**2**	
**SOMO**	**SOMO-1**
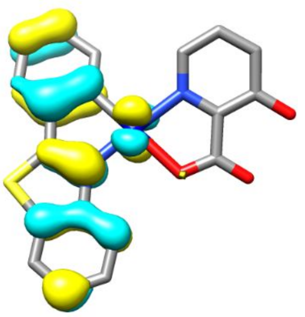	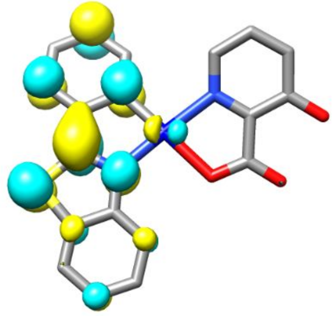
pbt 92%. OH-pic 3%. Pt 5%	pbt 84%. OH-pic 2%. Pt 15%
**$[2]_2$[a]**	
**SOMO**	**SOMO-1**
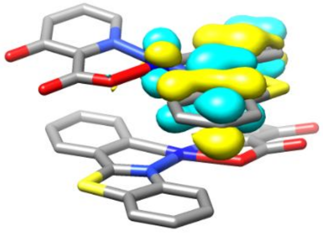	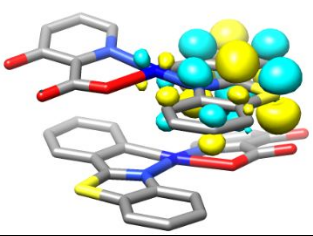
Molecule A: pbt(1) 86%. OH-pic(1) 4%. Pt(1) 4%<br>Molecule B: pbt(2) 4%. OH-pic(2) 0%. Pt(2) 1%	Molecule A: pbt(1) 82%. OH-pic(1) 2%. Pt(1) 13%<br>Molecule B: pbt(2) 2%. OH-pic(2) 0%. Pt(2) 1%
**$[2]_3$[a]**	
**SOMO**	**SOMO-1**
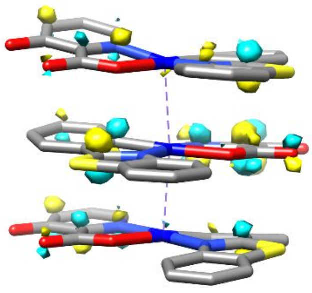	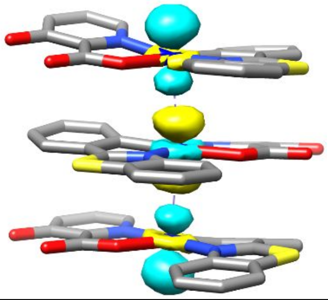
Molecule A: pbt(1) 14%. OH-pic(1) 10%. Pt(1) 5%<br>Molecule B: pbt(2) 17%. OH-pic(2) 23%. Pt(2) 5%<br>Molecule C: pbt(3) 10%. OH-pic(3) 13%. Pt(3) 4%	Molecule A: pbt(1) 3%. OH-pic(1) 2%. Pt(1) 25%<br>Molecule B: pbt(2) 3%. OH-pic(2) 1%. Pt(2) 35%<br>Molecule C: pbt(3) 3%. OH-pic(3) 2%. Pt(3) 25%

**$[2]_4$**[a]	
**SOMO**	**SOMO-1**
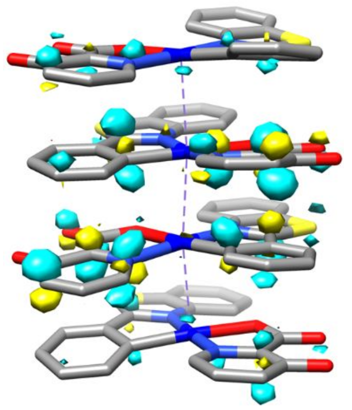	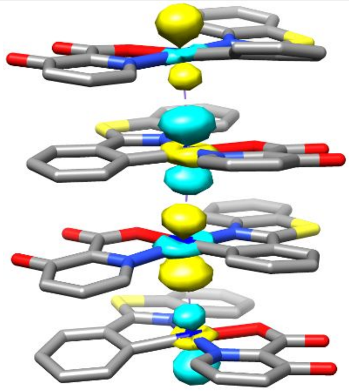
Molecule A: pbt(1) 5%. OH-pic(1) 7%. Pt(1) 2%<br>Molecule B: pbt(2) 13%. OH-pic(2) 18%. Pt(2) 4%<br>Molecule C: pbt(3) 13%. OH-pic(3) 18%. Pt(3) 4%<br>Molecule D: pbt(4) 5%. OH-pic(4) 7%. Pt(4) 2%	Molecule A: pbt(1) 2%. OH-pic(1) 1%. Pt(1) 14%<br>Molecule B: pbt(2) 3%. OH-pic(2) 1%. Pt(2) 29%<br>Molecule C: pbt(3) 3%. OH-pic(3) 1%. Pt(3) 29%<br>Molecule D: pbt(4) 2%. OH-pic(4) 1%. Pt(4) 14%
**$[2]_2$**[b]	
**SOMO**	**SOMO-1**
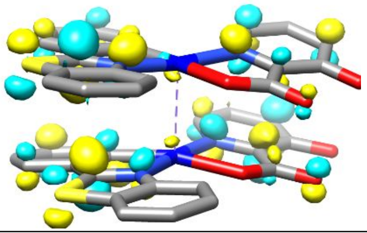	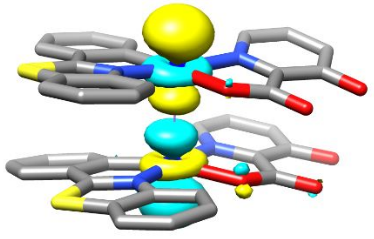
Molecule A: pbt(1) 31%. OH-pic(1) 13%. Pt(1) 5%<br>Molecule B: pbt(2) 25%. OH-pic(2) 20%. Pt(2) 5%	Molecule A: pbt(1) 5%. OH-pic(1) 2%. Pt(1) 43%<br>Molecule B: pbt(2) 5%. OH-pic(2) 2%. Pt(2) 43%
**$[2]_3$**[b]	
**SOMO**	**SOMO-1**
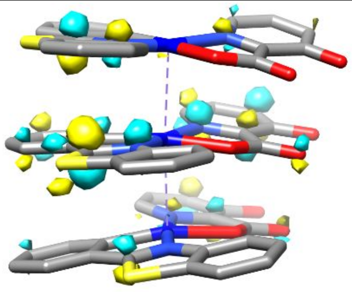	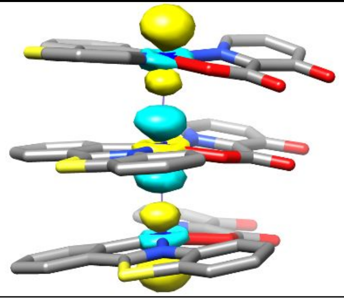
Molecule A: pbt(1) 15%. OH-pic(1) 7%. Pt(1) 3%<br>Molecule B: pbt(2) 26%. OH-pic(2) 18%. Pt(2) 4%	Molecule A: pbt(1) 3%. OH-pic(1) 2%. Pt(1) 25%<br>Molecule B: pbt(2) 3%. OH-pic(2) 1%. Pt(2) 35%

Molecule C: pbt(1) 11%. OH-pic(1) 12%. Pt(1) 3%	Molecule C: pbt(1) 3%. OH-pic(1) 2%. Pt(1) 26%
**$[2]_4$[b]**	
**SOMO**	**SOMO-1**
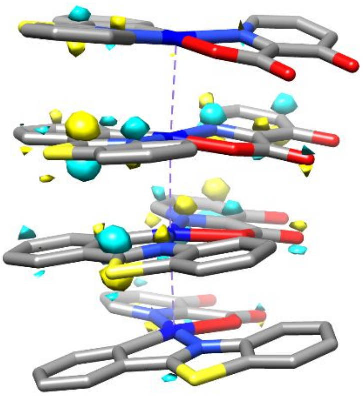	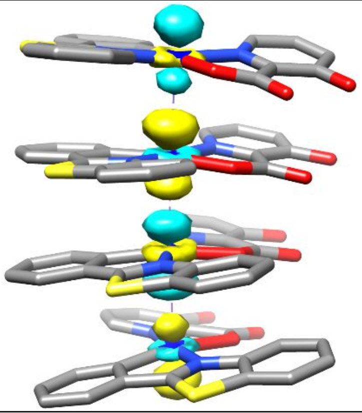
Molecule A: pbt(1) 8%. OH-pic(1) 4%. Pt(1) 2%<br>Molecule B: pbt(2) 20%. OH-pic(2) 13%. Pt(2) 4%<br>Molecule C: pbt(3) 17%. OH-pic(3) 15%. Pt(3) 4%<br>Molecule D: pbt(4) 5%. OH-pic(4) 7%. Pt(4) 2%	Molecule A: pbt(1) 2%. OH-pic(1) 2%. Pt(1) 14%<br>Molecule B: pbt(2) 3%. OH-pic(2) 1%. Pt(2) 28%<br>Molecule C: pbt(3) 3%. OH-pic(3) 1%. Pt(3) 28%<br>Molecule D: pbt(4) 2%. OH-pic(4) 1%. Pt(4) 15%

[a] Head-to-Tail, [b]Head-to-Head Structure

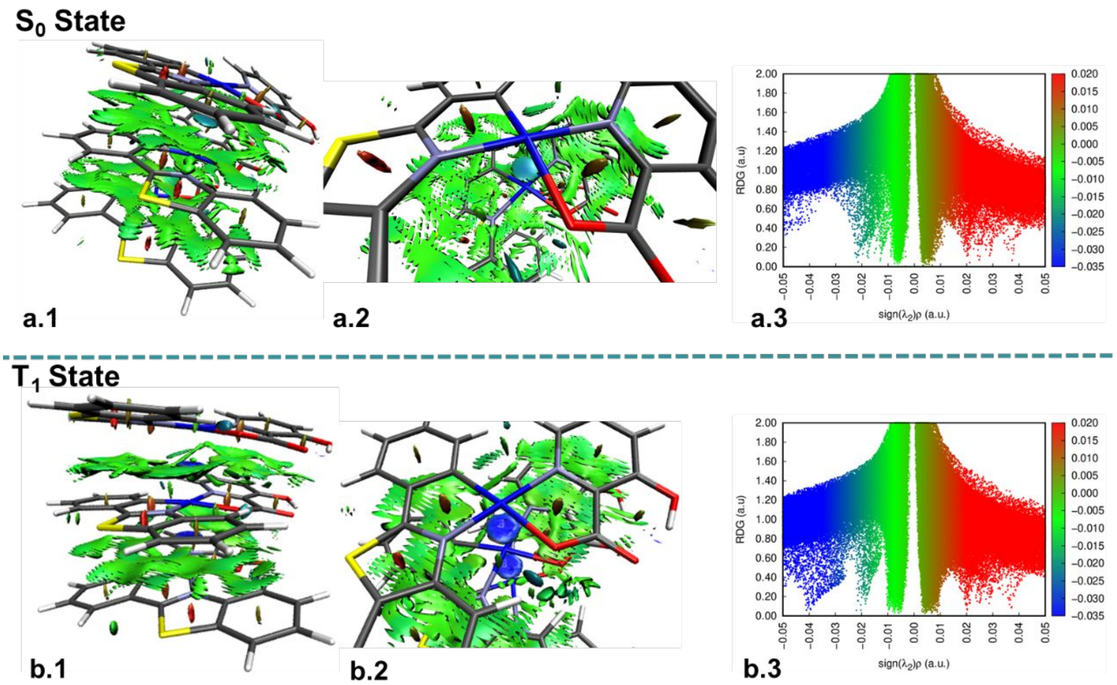

Figure S20. NCI analysis **[2]$_3$^{b}** (Head-to-Head) S_0 and T_1 optimized structures at level B3LYP-D3BJ (isovalue 0.3 a.u.).

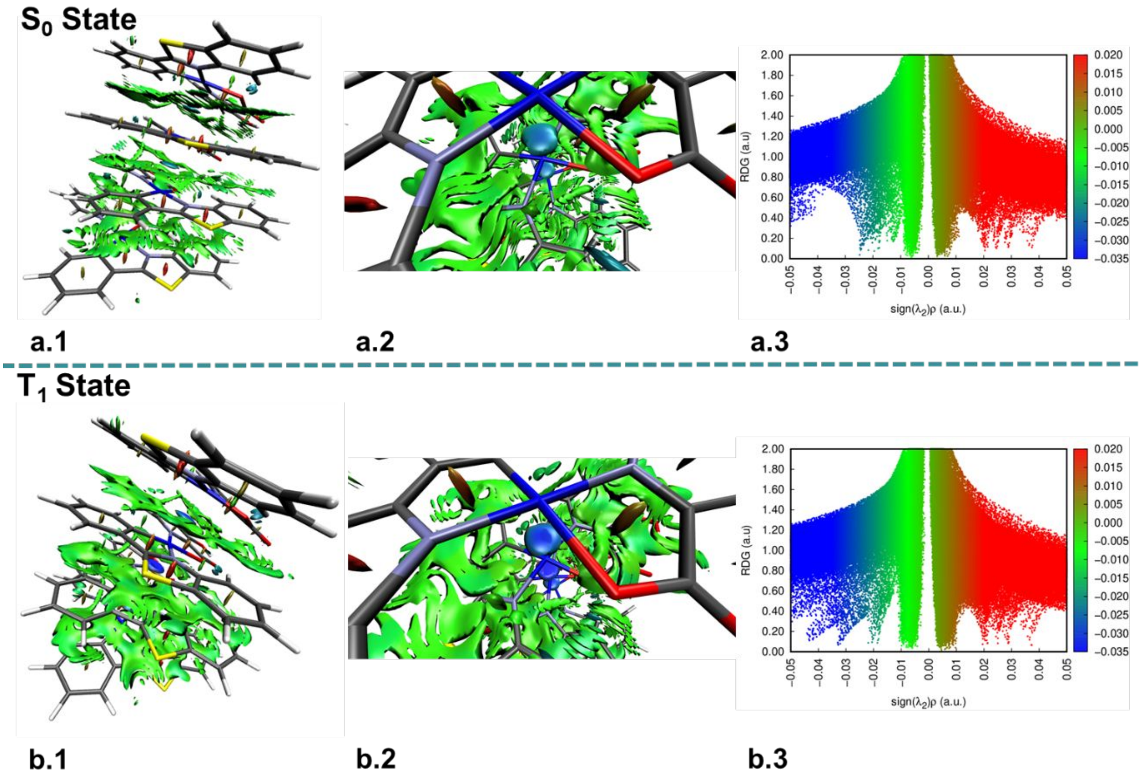

Figure S21. NCI analysis **[2]$_4$^{b}** (Head-to-Head) S_0 and T_1 optimized structures at level B3LYP-D3BJ (isovalue 0.3 a.u.).
